# Optimal dose of aquatic exercise for improving muscle strength in older adults: a Bayesian model-based meta-analysis

**DOI:** 10.3389/fpubh.2025.1699018

**Published:** 2025-12-11

**Authors:** Yifei Wang, Jin Zhao, Mingchen Gao, Xiaobin Wu

**Affiliations:** 1 School of Physical Education, Chengdu Sport University, Chengdu, Sichuan, China; 2 Sports Teaching and Research Department, Lanzhou University, Lanzhou, Gansu, China

**Keywords:** aquatic exercise, muscle strength, older adults, dose–response analysis, meta-analysis

## Abstract

**Objective:**

Amid global population aging, the progressive loss of muscle strength is a critical challenge compromising the functional independence of older adults. This study aimed to systematically quantify the dose–response relationship between various doses of aquatic exercise (frequency, session duration, total weekly duration, period, and intensity) and muscle strength in healthy older adults through a Bayesian model-based meta-analysis, in order to identify the optimal exercise protocol and inform precision exercise prescription.

**Methods:**

PubMed, Embase, Cochrane Library, Web of Science, China National Knowledge Infrastructure (CNKI), and Wan Fang Data were systematically searched for RCTs on aquatic exercise and older adults’ muscle strength up to March 2025. A conventional meta-analysis evaluated overall effects, followed by a Bayesian model-based dose–response meta-analysis using cubic restricted splines to quantify non-linear relationships. Standardized mean difference (SMD, with 95% CI/CrI) served as the effect measure.

**Results:**

A total of 13 RCTs (*n* = 531) were included. Overall, aquatic exercise significantly improved muscle strength (SMD = 0.56, 95% CI: 0.39–0.74, *p* < 0.0001). Dose–response analysis revealed cumulative trends for intervention period (peaking at 24 weeks, SMD = 0.65), frequency (2–3 sessions/week for significant gains), and total weekly duration (plateauing after 200 min, with significant effect at 100 min, SMD = 0.58). Conversely, session duration (optimal 30–45 min, peaking at 40 min, SMD = 0.62) and intensity (optimal Borg RPE 10–12, SMD = 0.45) showed inverted U-shaped relationships.

**Conclusion:**

Aquatic exercise is an effective strategy for improving muscle strength in healthy older adults, with its benefits demonstrating significant non-linear dose–response relationships. To maximize efficacy, the optimal recommended protocol consists of 2–3 sessions per week, each lasting approximately 40 min, at a moderate-to-high intensity (Borg RPE 10–12), and should be adopted as a long-term strategy. However, substantial heterogeneity and evidence of publication bias suggest cautious interpretation of pooled effects.

**Systematic review registration:**

Identifier: CRD420250654651.

## 1 Introduction

With the accelerating trend of global population aging, the decline in muscle strength has emerged as a pressing public health concern. For older adults, independence is closely associated with muscle strength, which is directly linked to their ability to perform daily activities (1). With advancing age, muscle weakness compromises skeletal support and visceral functions (2), creating a vicious cycle of “muscle loss → reduced activity → further muscle loss,” which substantially impairs quality of life (3). Evidence shows that in individuals aged over 60 years, muscle mass decreases by 1–2% annually (4), and this decline is the primary cause of reduced strength. Moreover, diminished strength has been shown to be significantly associated with the ability to perform basic activities of daily living (5, 6).

Among the non-pharmacological strategies to counteract age-related functional decline, regular physical exercise has been widely validated by evidence-based research. Systematic studies demonstrate that structured training programs can substantially improve multiple functional outcomes in older adults—such as balance, agility, cardiorespiratory endurance, and muscle strength—through both neuromuscular adaptations and metabolic regulation (7). Aquatic exercise stands out as an effective strategy for enhancing muscle strength (8–10). As a low-impact training modality, aquatic exercise reduces mechanical loading due to the buoyancy of water, thereby minimizing compressive stress on joints and muscles, lowering exercise-related risks, and enhancing overall health. Because water has approximately 800 times greater density than air and exerts viscosity, movements performed in water encounter greater resistance, requiring more muscular involvement and greater concentric force production (11). Additionally, compared with land-based exercise, aquatic environments provide a safe setting for low-intensity training (12). Even frail older adults unable to exercise safely on land may benefit from aquatic programs, while the supportive and enjoyable environment also promotes adherence (13). Nevertheless, the effects of aquatic exercise on muscle strength in older adults remain inconsistent across studies.

Experimental evidence suggests that aquatic training can enhance muscle strength, with high-intensity aquatic protocols effectively improving both upper- and lower-limb strength in older women (14). Evidence from systematic reviews and meta-analyses further indicates that aquatic exercise improves functional performance in both younger and older adults (15, 16), reduces blood pressure (17), and increases maximal oxygen uptake (18). The use of resistance equipment to regulate exercise load during aquatic sessions may optimize strength gains in older adults (19). Despite growing recognition of its benefits, the optimal dosage and characteristics of aquatic exercise for mitigating age-related muscle decline remain to be clarified (20).

To date, only one meta-analysis has preliminarily addressed the effects of aquatic exercise on muscle strength in healthy older adults. Prado et al. confirmed that aquatic exercise is potentially effective for improving muscle strength (21). However, their conclusions also revealed substantial limitations and inconsistencies: while many individual studies reported positive findings, their meta-analysis showed no significant effects on lower-limb isometric strength compared with controls, with high heterogeneity across studies. This indicates that a simple pooled effect size may be insufficient to resolve the inconsistencies in current evidence.

Given these methodological shortcomings and the high heterogeneity of previous reviews, the specific effects of aquatic exercise on physical function in healthy older adults, as well as the optimal exercise dose for maximizing benefits, remain uncertain. Therefore, we conducted a systematic review and meta-analysis of randomized controlled trials (RCTs) to evaluate the effects of different aquatic exercise doses on muscle strength in older adults. The findings are expected to provide evidence-based guidance for designing exercise interventions and training programs for this population.

## 2 Methods

### 2.1 Experimental approach to the problem

This study followed the Preferred Reporting Items for Systematic Reviews and Meta-Analyses (PRISMA) statement guidelines (22), and the study protocol is registered in PROSPERO (CRD420250654651).

### 2.2 Study selection and search strategies

Two reviewers independently and in a blinded, duplicate fashion conducted systematic searches of PubMed, Embase, Cochrane Library, Web of Science, China National Knowledge Infrastructure (CNKI), and Wan Fang Data from database inception through March 30, 2025. The search was restricted to articles published in English or Chinese. In addition, we screened the reference lists of published systematic reviews and meta-analyses to ensure comprehensive coverage. The detailed search strategies for all databases including search terms, dates, and procedures are provided in Supplementary file 1.

### 2.3 Eligibility criteria

Based on the PICOS framework and in alignment with our study objectives, the following inclusion and exclusion criteria were established (23):

Inclusion criteria: (1) Participants: studies including healthy older adults aged ≥60 years, regardless of sex, training level, or prior experience with aquatic exercise. (2) Interventions: studies must include at least one experimental group implementing upright aquatic aerobic exercise (e.g., water resistance, aquatic aerobic, or combined training) for a duration of ≥4 weeks. (3) Control group: a no-exercise control or maintenance of usual activities. (4) Outcome measures: studies must report at least one land-based measure of muscle strength or power-related indicators (e.g., 1RM, peak torque, power, jump height). (5) Study design: only randomized controlled trials (RCTs) were included.

Exclusion criteria: (1) Study design: non-RCT studies were excluded. (2) Participants: studies including participants with major illnesses or in post-operative recovery were excluded. (3) Intervention type: only studies involving upright aquatic exercise were included; studies that were incomplete or had missing outcome data were excluded. (4) Publication type: conference abstracts, dissertations, or other unpublished studies were not included.

### 2.4 Study selection and data extraction

Two reviewers independently screened records in EndNote X9 according to the predefined inclusion and exclusion criteria; any discrepancies were resolved through discussion with a third reviewer until consensus was reached. Data were extracted on the following items: (1) study characteristics (title, first author, year of publication, country, sample size); (2) participant characteristics (age, sex, health status); (3) intervention details (type of aquatic exercise, intervention duration, session length, frequency, intensity); and (4) outcome measures (assessment tools and data), including upper- and lower-limb strength indices.

To minimize the influence of subjective factors on data conversion, the following methods were applied: (1) for studies reporting fixed values for frequency, duration, or intensity, the original data were retained; (2) for studies reporting a range of frequency, duration, or intensity, the mean value of the range was used; (3) for studies reporting exercise intensity in different formats, a standard conversion to the Borg RPE scale was applied. Subgroup analyses were conducted for influencing factors such as training elements, exercise types, and different outcome measures, aiming to explore the dose–response effects of various aspects of aquatic exercise on muscle strength. Studies with missing key dose variables that could not be estimated were excluded from the corresponding subgroup or dose–response analyses.

### 2.5 Risk of bias

Two researchers independently assessed the quality of the included studies using the PEDro scale, which is based on standards from the Physiotherapy Evidence Database (PEDro). Overall, the quality of the included studies was moderate, with PEDro scores ranging from 3 to 7; four studies scored above 6 (Supplementary file 3).

**Figure 1:**
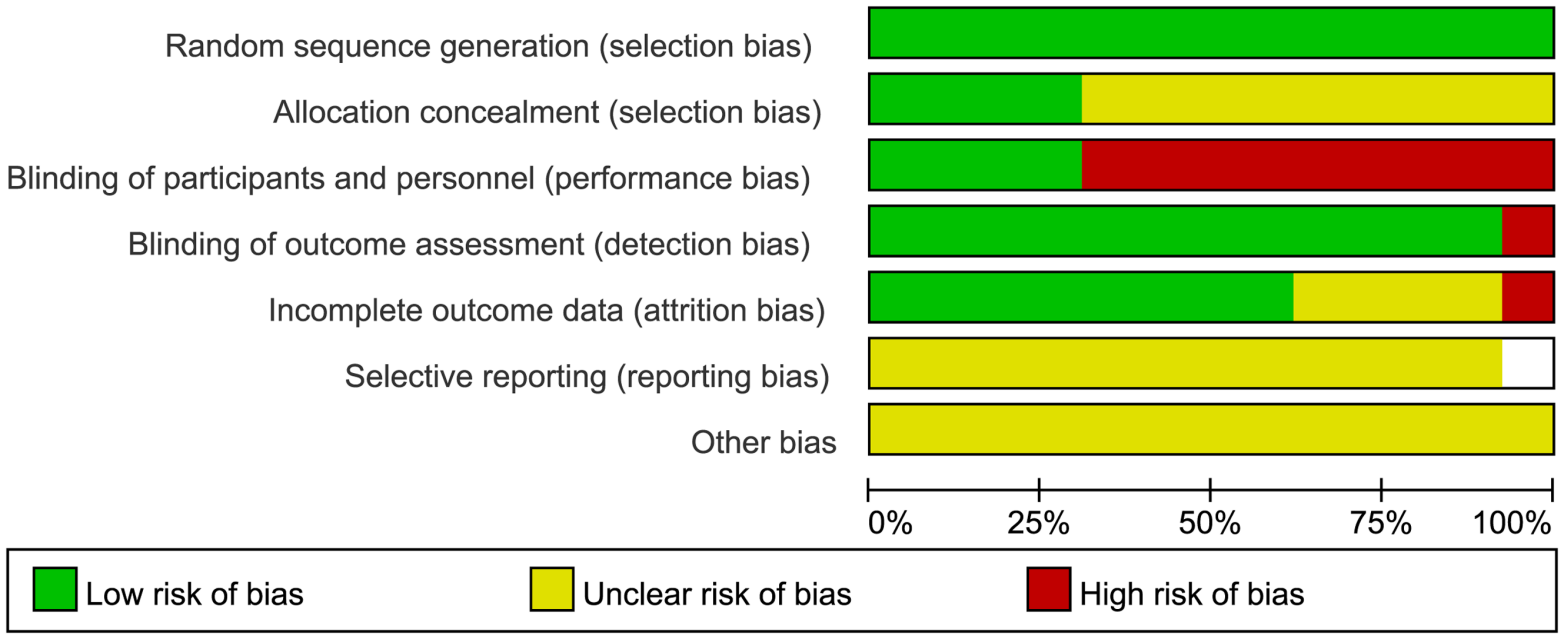
Risk of bias of the included literature.

**Figure 2:**
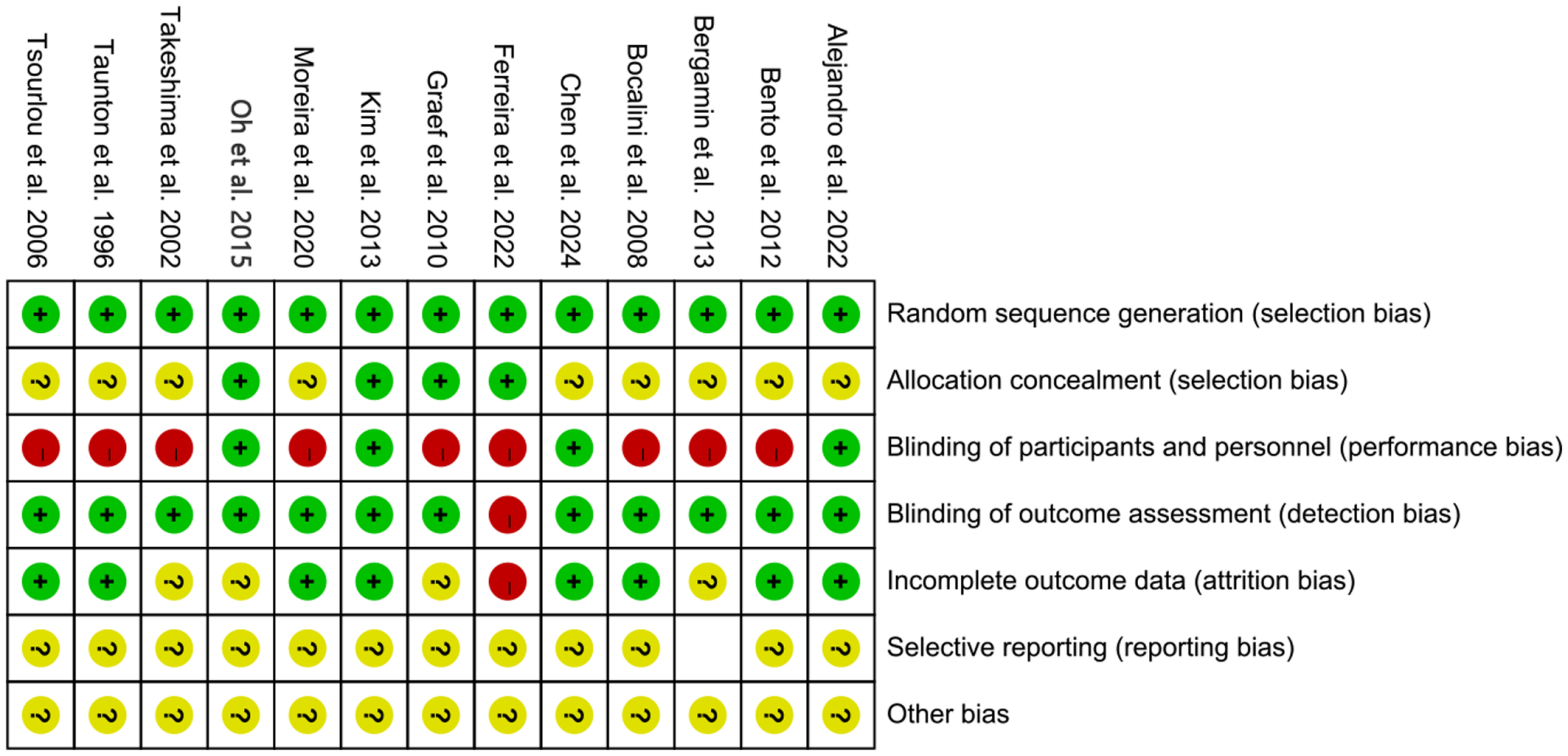
Summary of risk of bias of the included literature. “−” indicates high risk of bias; “?” indicates unclear risk of bias; “+” indicates low risk of bias.

The two researchers also independently evaluated the risk of bias using the Cochrane Risk of Bias tool. Discrepancies were resolved by a third researcher. The results of the risk of bias assessment are shown in Figures 1, 2. Thirteen studies reported the specific methods for generating random sequences, including random number tables and computer-based randomization. Nine studies did not report whether blinding was implemented (9, 19, 24–30), and one study experienced sample loss due to COVID-19 (24). No studies showed evidence of selective reporting. Moreover, due to the nature of exercise interventions, blinding participants and personnel is inherently challenging, which may have influenced the methodological quality of the included studies. A detailed risk of bias table is provided in Supplementary file 3.

### 2.6 Statistical analysis

#### 2.6.1 For meta-analysis

All meta-analyses were conducted in the R statistical environment (version 4.5.0)^1^ using the tidyverse and meta packages. Considering that the included studies may have used different measurement scales, the standardized mean difference (SMD) was chosen as the common effect size, with Hedges’ g applied to correct for small-sample bias. Given the potential heterogeneity among studies, a random-effects model was used for data pooling, with between-study variance estimated via the robust restricted maximum likelihood (REML) method. To improve the precision of confidence intervals, the Hartung–Knapp–Sidik–Jonkman (HKSJ) adjustment was applied. Potential publication bias was assessed using enhanced contour-enhanced funnel plots (31), which incorporated contour lines for statistical significance levels (*p* = 0.01, 0.05, 0.1) to help distinguish between asymmetry due to publication bias and other causes. All statistical tests were two-tailed, and *p* < 0.05 was considered statistically significant.

#### 2.6.2 For dose–response analysis

For the dose–response meta-analysis, we employed the MBNMAdose package (version 4.2.2, see text footnote 1) along with the gemtc and rjags packages in R (version 4.5.0) to perform a random-effects Bayesian model-based network meta-analysis (MBNMA) (32). This approach summarized the relationship between aquatic exercise dose and changes in muscle strength. We first plotted the treatment-level network to verify connectivity—a key assumption in network dose–response meta-analysis—since lack of connectivity can result in low statistical power and misleading results (33). Samples were generated using a Markov chain, and model convergence was assessed using the Potential Scale Reduction Factor (PSRF). The data were then analyzed using both a consistency model and an unrelated mean effects model, and their performance was compared in terms of deviance, the number of estimated parameters, and the deviance information criterion (DIC) to evaluate model fit. Similarity in these metrics was interpreted as evidence of good consistency (34). Transitivity was assessed using the node-splitting method in MBNMA, which partitions the contribution of a given treatment comparison into direct and indirect evidence. Comparable effects between the two were taken as evidence of good transitivity (35). To explore the functional relationship between exercise dosage and muscle strength in older adults, we fitted multiple non-linear functional models to the data, including the Emax function, restricted cubic splines, linear models, non-parametric dose–response models, and exponential dose–response functions. All models were compared using fit indices and corresponding deviance plots (36). Effect sizes for muscle strength outcomes were interpreted based on Cohen’s guidelines (37): small effect (SMD ≈ 0.2), moderate effect (SMD ≈ 0.5), and large effect (SMD ≥ 0.8).

## 3 Results

### 3.1 Description of the studies

**Figure 3:**
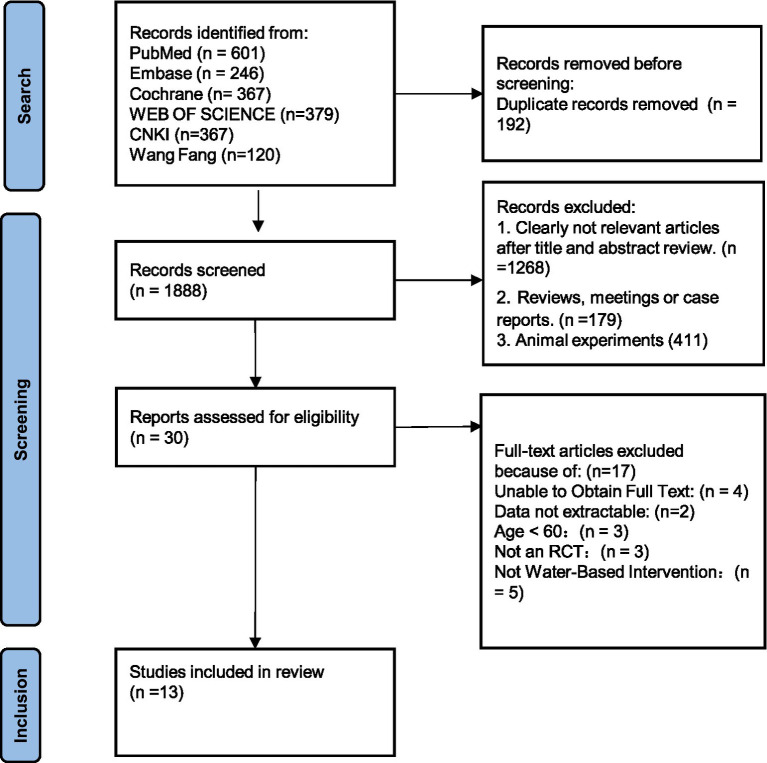
Flow chart of literature search.

A total of 2,080 records were initially identified. After removing duplicates, 1,888 records remained. Screening of titles and abstracts yielded 30 potentially eligible articles, and after full-text assessment, 13 studies met the inclusion criteria. These studies involved a total of 531 participants, including 287 in the intervention groups and 244 in the control groups. The study selection process is illustrated in Figure 3, and the characteristics of the included studies are provided in Supplementary file 1.

The sample sizes of the included studies ranged from 15 to 120 participants, with a median of 38. The mean age of participants ranged from 60 to 75 years, with a median of 69.3 years. The publication years spanned from 1996 to 2024, with a median year of 2014. Regarding aquatic exercise interventions, the reported duration ranged from 10 to 24 weeks (median: 12 weeks), training frequency ranged from 2 to 3 sessions per week (median: 3 sessions), and the duration of each session ranged from 45 to 70 min (median: 60 min).

### 3.2 Meta-analysis

We pooled data from the 13 included studies to evaluate the overall effect of aquatic exercise on muscle strength in older adults. The heterogeneity test indicated substantial variability in effect sizes across studies (*p* < 0.0001; *I*^2^ = 78.6%). Therefore, a random-effects model was applied for data synthesis.

**Figure 4:**
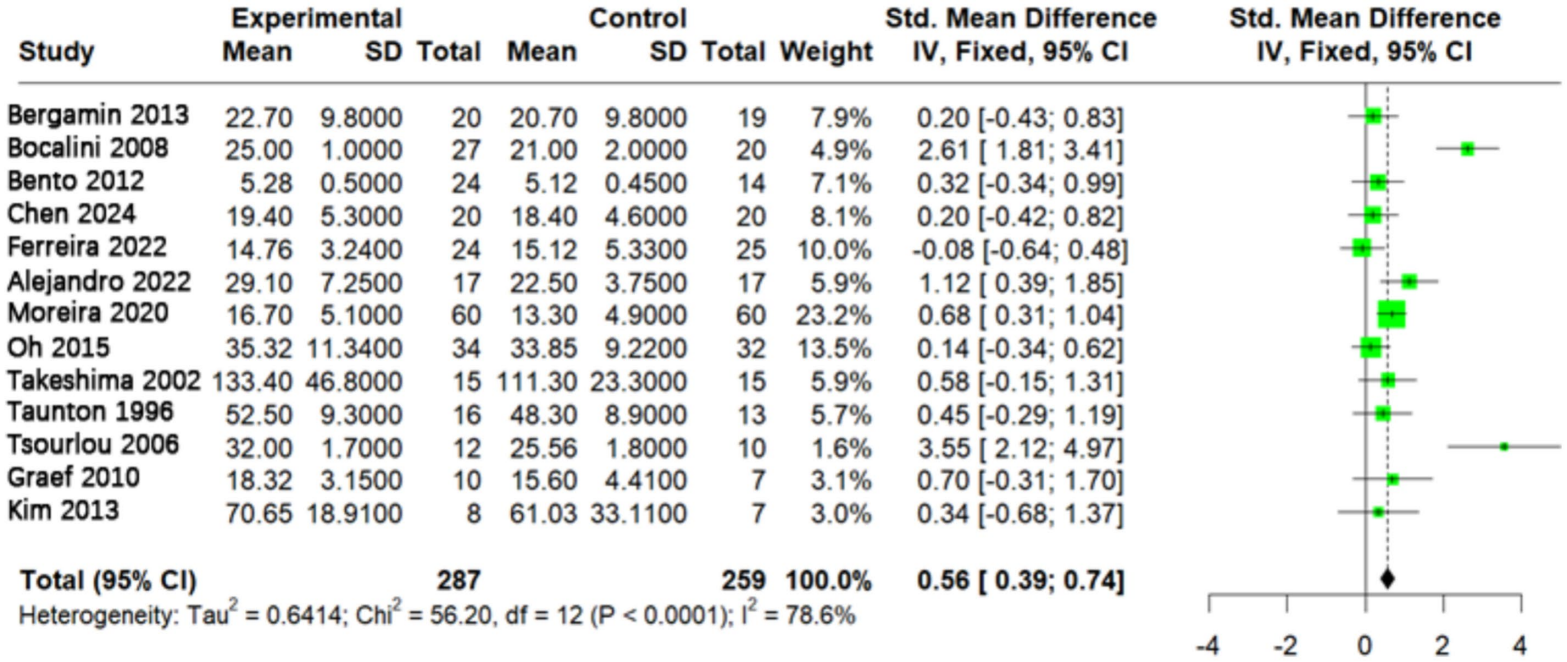
Effect of aquatic exercise on muscle strength in older adults. Aquatic exercise significantly improved muscle strength in older adults (*p* < 0.05).

The forest plot analysis revealed that, compared with control groups, aquatic exercise significantly improved muscle strength in older adults, with a pooled SMD of 0.56 (95% CI: 0.39–0.74; *p* < 0.0001). This finding indicates a statistically significant, moderate positive effect of aquatic exercise on muscle strength (Figure 4). To explore the source of the high heterogeneity, subgroup analyses were performed based on key intervention characteristics.

### 3.3 Effect of different aquatic exercise frequencies on muscle strength in older adults

**Figure 5:**
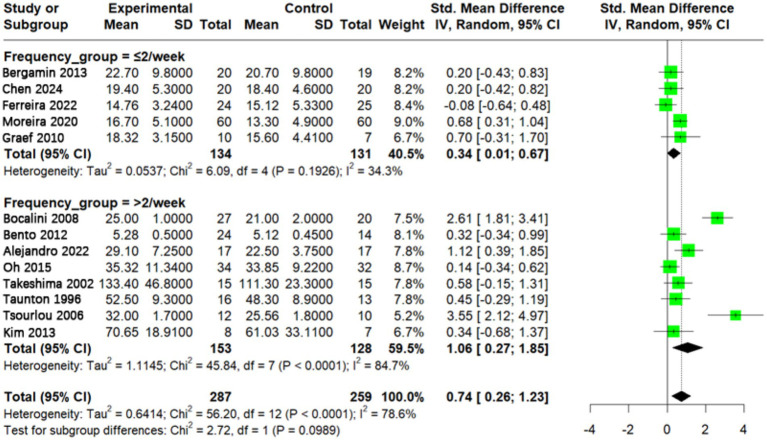
Effect of aquatic exercise frequency on muscle strength in older adults. Subgroup analysis showed that both exercise frequencies of ≤2 sessions/week and >2 sessions/week significantly improved muscle strength in older adults (*p* < 0.05). However, higher exercise frequency did not show a statistically significant difference compared with lower frequency (*p* > 0.05).

A total of 13 studies were included in the meta-analysis examining the effects of aquatic exercise frequency on older adults. Among them, five studies reported a training frequency of ≤2 sessions per week (19, 24, 27, 38, 39), while eight studies reported >2 sessions per week (9, 25, 26, 28–30, 40, 41). The subgroup performing interventions more than twice per week demonstrated a substantially larger effect size (SMD = 1.06, 95% CI: 0.27–1.85) compared with the subgroup performing interventions ≤2 times per week (SMD = 0.34, 95% CI: 0.01–0.67). Although the between-group difference did not reach statistical significance (*p* > 0.05), the point estimates suggest that higher-frequency interventions may yield greater improvements in muscle strength (Figure 5).

### 3.4 Effect of different aquatic exercise session time on muscle strength in older adults

**Figure 6:**
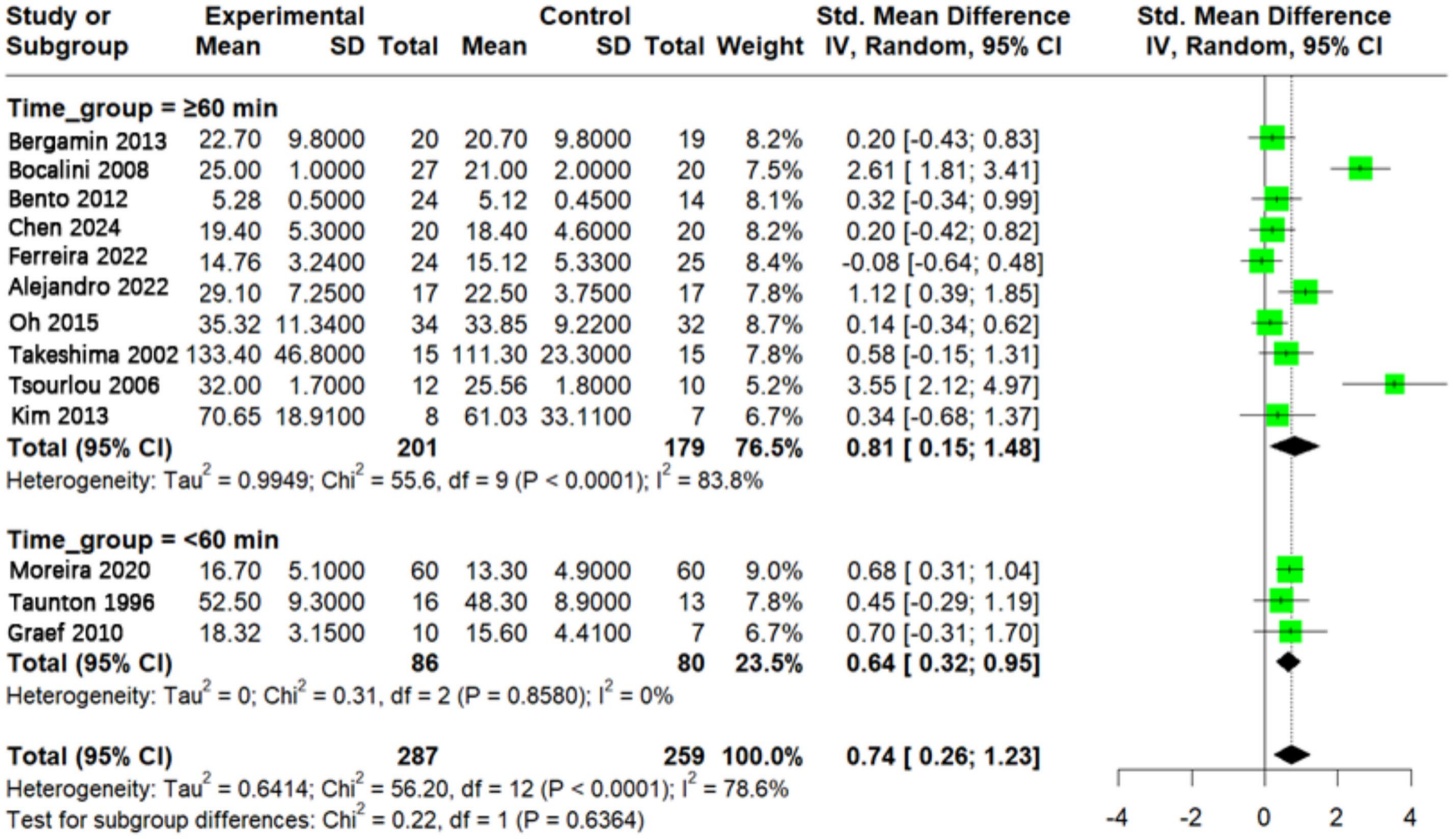
Effect of aquatic exercise time on muscle strength in older adults. Subgroup analysis showed that both interventions with a single session duration ≥60 min and <60 min significantly improved muscle strength in older adults (*p* < 0.05). However, longer exercise duration did not show a statistically significant difference in effect compared with shorter duration (*p* > 0.05).

To investigate the effects of session duration of aquatic exercise on muscle strength in older adults, a total of 13 studies were included in the meta-analysis. Among them, 10 studies reported single-session durations of ≥60 min (9, 24, 26–30, 38, 40, 41), while three studies reported durations of <60 min (19, 25, 39). Sessions lasting ≥60 min produced a larger effect size (SMD = 0.81, 95% CI: 0.15–1.48), while sessions <60 min yielded a moderate effect size (SMD = 0.64, 95% CI: 0.32–0.95). No statistically significant difference was found between the two subgroups (*p* > 0.05) (Figure 6).

### 3.5 Effect of different aquatic exercise intervention period on muscle strength in older adults

**Figure 7:**
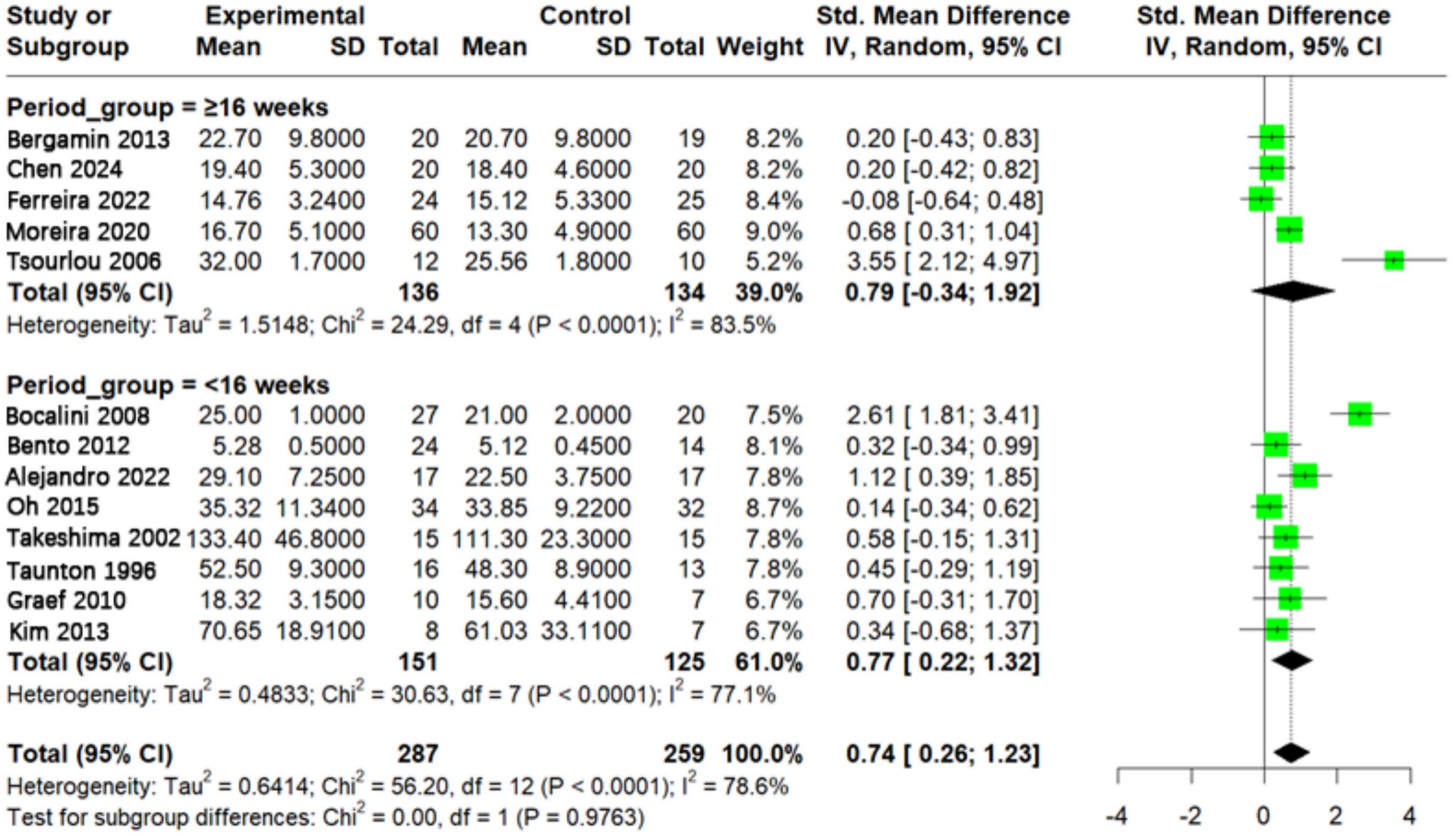
Effect of aquatic exercise intervention period on muscle strength in older adults. Subgroup analysis showed that interventions with a period of <16 weeks significantly improved muscle strength in older adults (*p* < 0.05), while interventions lasting ≥16 weeks did not reach statistical significance. No statistically significant difference in effect was found between the two groups (*p* > 0.05).

To examine the impact of intervention period, a total of 13 studies were included in the meta-analysis on the effects of aquatic exercise duration on older adults. Among them, five studies had an intervention period of ≥16 weeks (24, 27, 30, 38, 39), while eight studies had an intervention period of <16 weeks (9, 19, 25, 26, 28, 29, 40, 41). The analysis of intervention period showed that the ≥16-week subgroup had a pooled effect size of SMD = 0.79 (95% CI: −0.34 to 1.92), while the <16-week subgroup had SMD = 0.77 (95% CI: 0.22–1.32). The between-group difference was not statistically significant (*p* = 0.98), indicating that intervention duration was not a major source of heterogeneity. However, the ≥16-week subgroup had a wider confidence interval including zero and comprised only five studies, suggesting limited statistical evidence and greater uncertainty in effect estimates. Therefore, caution is warranted when interpreting long-term intervention effects (Figure 7).

### 3.6 Effect of different aquatic exercise intensities on muscle strength in older adults

**Figure 8:**
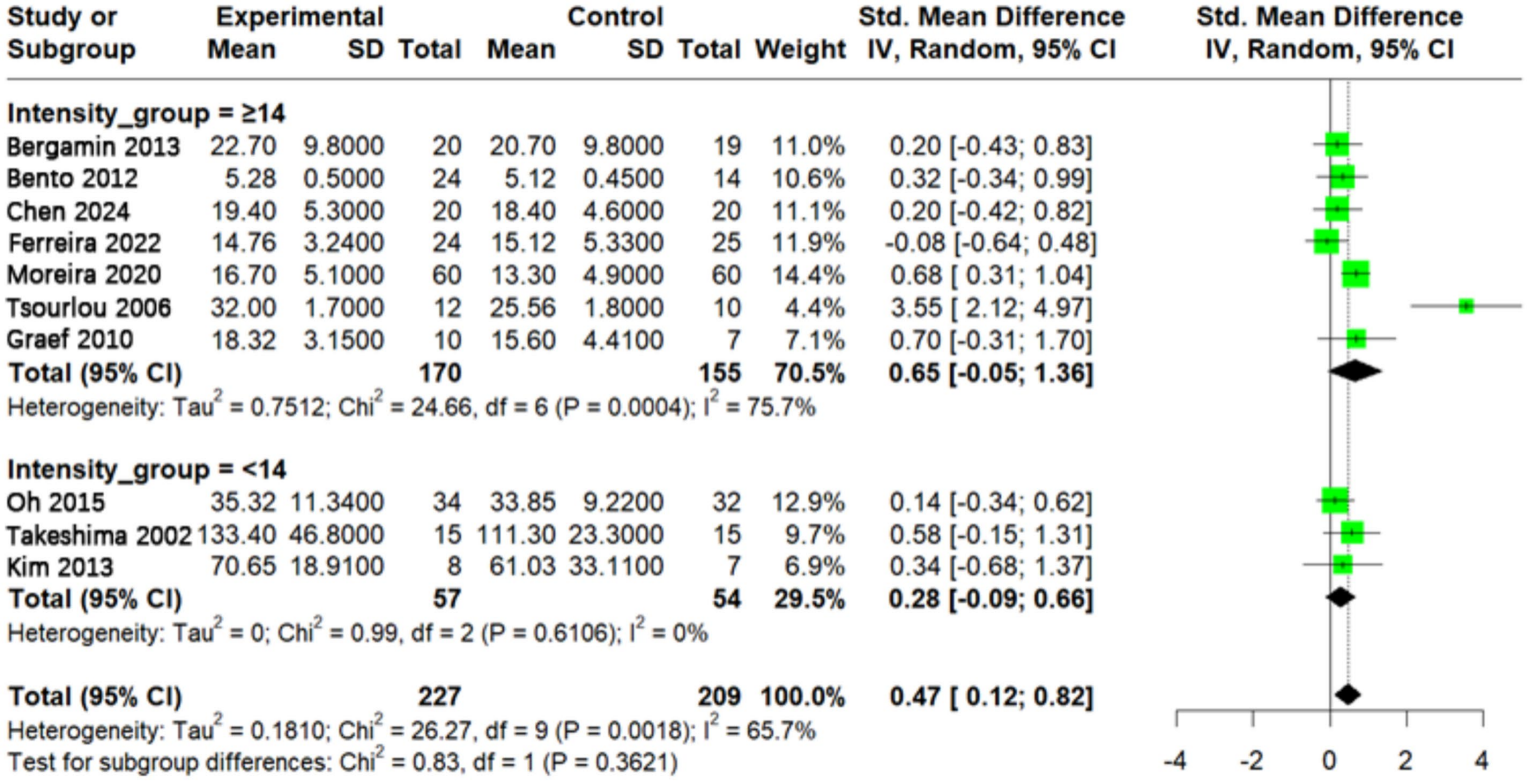
Effect of aquatic exercise intervention intensity on muscle strength in older adults.

A total of 10 studies reported the intensity of single-session aquatic exercise. Among these, seven studies involved moderate to high intensity (RPE ≥ 14) (9, 19, 24, 27, 30, 38, 39), while three studies were classified as low intensity (RPE < 14) (28, 29, 41). Interventions at higher intensity levels (RPE ≥ 14) showed a larger effect estimate (SMD = 0.65, 95% CI: −0.05 to 1.36), whereas low to moderate intensity interventions (RPE < 14) had a smaller effect size (SMD = 0.28, 95% CI: −0.09 to 0.66). The between-group difference was not statistically significant (*p* = 0.36), indicating insufficient evidence to confirm a definitive impact of intensity on muscle strength (Figure 8).

### 3.7 Effects of different aquatic exercise outcome measures on muscle strength in older adults

Thirteen studies reported outcomes using different measures. Among these, seven studies assessed specific muscle group strength using instruments (19, 24, 25, 27–29, 41), four studies assessed strength through tasks simulating activities of daily living (26, 27, 38, 39), and two studies evaluated both muscle strength and functional strength (9, 30).

**Figure 9:**
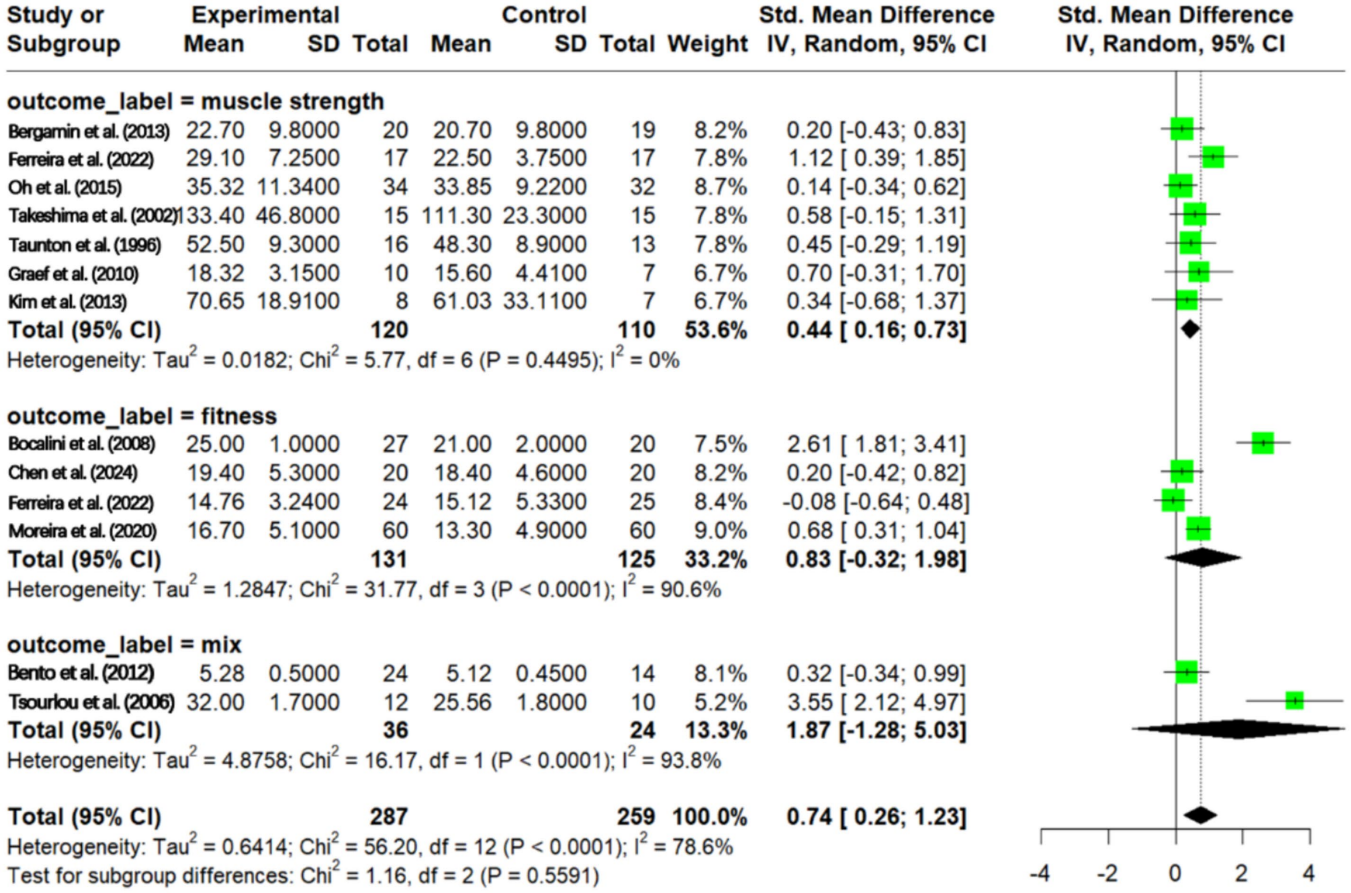
Effects of aquatic exercise outcome measures on muscle strength in older adults.

The results showed that in the direct muscle strength group, aquatic exercise had a significant positive effect on muscle strength (SMD = 0.44, 95% CI: 0.16–0.73), with very low heterogeneity within this subgroup. In the functional strength group, the pooled effect size was not statistically significant (SMD = 0.83, 95% CI: −0.32 to 1.98), and there was very high within-group heterogeneity (*I*^2^ = 90.6%). The mixed assessment group showed similarly high heterogeneity (*I*^2^ = 93.8%) (Figure 9).

### 3.8 Sensitivity analysis

**Figure 10:**
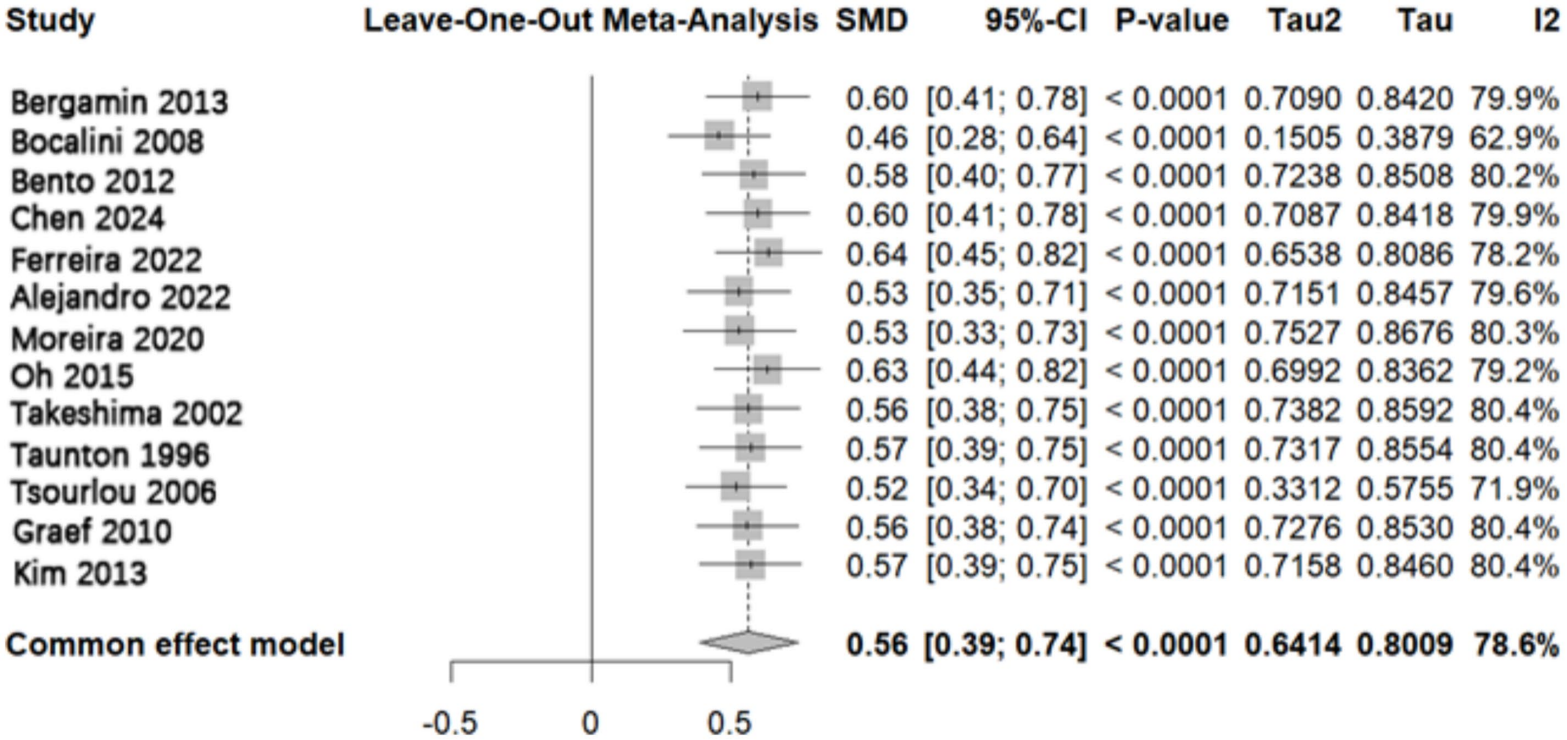
Sensitivity analysis of the effect of aquatic exercise on muscle strength in older adults. After sequentially omitting each study, the direction and statistical significance of the pooled effect size remained largely unchanged, and all values remained within the credible interval, indicating the robustness of the findings.

To assess the robustness of the meta-analysis results, a leave-one-out sensitivity analysis was conducted (Figure 10). The results showed that sequentially removing any single study yielded pooled SMDs ranging from 0.46 to 0.64, all remaining statistically significant (*p* < 0.05). These findings indicate that the overall conclusions of the meta-analysis were not driven by any individual study and demonstrate good robustness of the results.

### 3.9 Publication bias analysis

**Figure 11:**
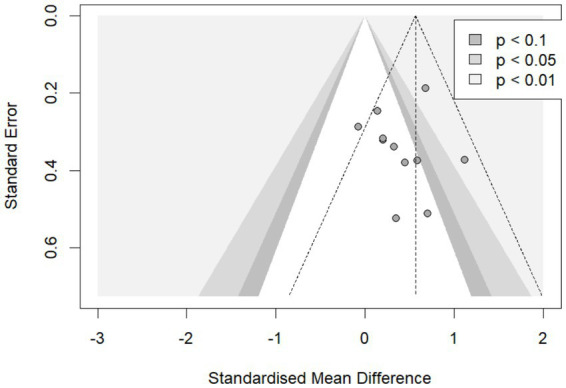
Funnel plot for publication bias of outcome measures. The funnel plot shows some asymmetry, suggesting potential publication bias. The horizontal axis represents the effect size expressed as standardized mean difference (SMD), and the vertical axis represents the standard error.

Potential publication bias was assessed using visual inspection of funnel plots and Egger’s linear regression test (Figure 11). The results showed asymmetry in the distribution of studies, suggesting the presence of some publication bias, which may be related to the relatively low quality and small sample sizes of some included studies. Although the traditional Egger’s test did not provide statistical evidence of bias (*p* = 0.1274), the observed funnel plot asymmetry prompted the use of the more robust Vevea & Hedges weight-function model for assessment (Supplementary file 10). The results confirmed significant publication bias among the included studies (likelihood ratio test, *p* = 0.0088), which may again be attributable to the lower quality and smaller sample sizes of certain studies.

### 3.10 Dose–response relationships

**Figure 12:**
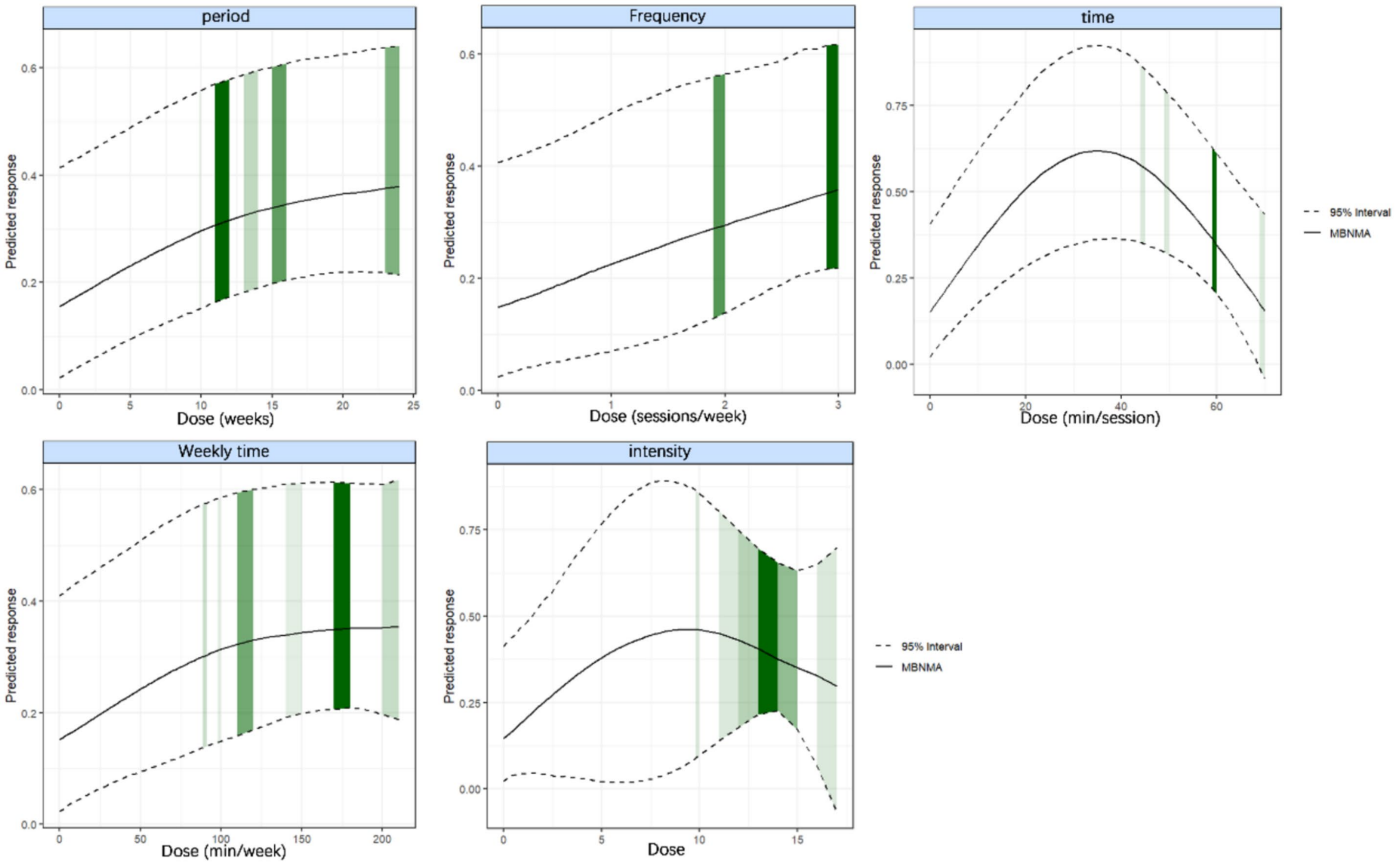
Dose–response relationship between different covariates and muscle strength changes in older adults. The horizontal axis represents the five exercise dose dimensions (intervention period, frequency, session duration, weekly total duration, and exercise intensity); the vertical axis represents the predicted response (SMD). The solid line indicates the predicted mean response at each dose level estimated by the model-based MBNMA. The dashed lines represent the 95% credible interval. The vertical green bands show the distribution and density of data from the included studies, with darker or wider bands indicating greater evidence supporting that specific dose level.

To explore the relationship between different dose dimensions of aquatic exercise and intervention effects, dose–response analyses were conducted for five key dose variables: exercise frequency, total weekly duration, single-session duration, intervention period, and intensity. Based on DIC values and model flexibility, restricted cubic splines were ultimately selected to uniformly analyze and visualize the nonlinear relationships across all dose dimensions. Following the best fit and biological plausibility, three knots were placed at the 10th, 50th, and 90th percentiles of each dose variable (42) (Supplementary file 5). Node-splitting analyses indicated good consistency across all dose networks (*p* > 0.05). Furthermore, all reported dose–response models passed convergence diagnostics (R-hat < 1.05). The dose–response curves for each dose dimension are shown in Figure 12.

The results revealed two distinct nonlinear trends in the dose–response relationship for older adults’ aquatic strength training. Intervention period (weeks), exercise frequency (sessions/week), and total weekly duration (min/week) exhibited continuously increasing trends that plateaued with higher doses. In contrast, session duration (min/session) and exercise intensity displayed an inverted U-shaped relationship, indicating differing responsiveness of older adults to various training dosages.

As the intervention period increased, the predicted SMD rose steadily, with a minimal significant effect observed at 12 weeks (SMD = 0.55; 95% CrI 0.28–0.62) and the maximum effect achieved at 24 weeks (SMD = 0.65; 95% CrI 0.40–0.66). Exercise frequency was similarly positively correlated with muscle strength improvement in an approximately linear manner; two sessions per week already produced significant gains (SMD = 0.56; 95% CrI 0.22–0.58), while three sessions per week resulted in a slight further increase (SMD = 0.62; 95% CrI 0.24–0.62). Total weekly training duration reached significant effects at 100 min/week (SMD = 0.58; 95% CrI 0.34–0.60) and achieved maximal gains at 200 min/week (SMD = 0.62; 95% CrI 0.38–0.62), with limited marginal improvement beyond this point.

In contrast, session duration and exercise intensity followed an inverted U-shaped pattern. The peak effect of session duration occurred at 40 min (SMD = 0.62; 95% CrI 0.32–0.82), suggesting an optimal range of 30–45 min. Exercise intensity showed its peak effect within a Borg 10–12 range (SMD = 0.45; 95% CrI 0.18–0.46), with the minimum significant threshold at 12 (SMD = 0.30; 95% CrI 0.05–0.35), suggesting that moderate-to-high intensity within this range is the most effective practice.

## 4 Discussion

This systematic review aimed to comprehensively analyze the effects of aquatic exercise on muscle strength in healthy older adults and, through dose–response analysis, determine the optimal exercise dose. Thirteen studies published between 1996 and 2024 were included, all of which assessed at least one muscle strength outcome after the intervention. The meta-analysis of these 13 studies indicated that aquatic exercise can effectively improve muscle strength in healthy older adults (SMD = 0.56, 95% CI: 0.39–0.74, *p* < 0.0001), which is consistent with previous systematic review conclusions. To our knowledge, this study is the first to comprehensively analyze the dose–response relationship between aquatic exercise and muscle strength in older adults.

Overall, aquatic exercise demonstrated a significant positive effect on muscle strength. The water environment, through its turbulent effects, provides an unstable setting that challenges the neuromuscular system more than typically static land-based interventions. This promotes more frequent engagement of core and stabilizing muscles in movement control and enhances proprioceptive feedback, thereby improving overall muscle force output (43). This study specifically limited inclusion to upright aquatic exercises, as this posture requires participants to continuously counteract buoyancy and water perturbations to maintain upright balance, generating substantial training benefits while also reflecting functional movement patterns relevant to older adults’ daily activities. The pooled analysis showed high heterogeneity (*I*^2^ = 78.6%), indicating considerable variability in effect sizes among the included studies, suggesting that a simple overall effect may not fully capture the complex dose–response relationships.

To explore the sources of heterogeneity, subgroup analyses were conducted. The results indicated that subgroups with higher frequency, longer single-session duration, and greater intensity generally exhibited larger effects. However, these higher-dose subgroups also demonstrated greater heterogeneity, and the between-group differences did not reach statistical significance (*p* > 0.05). For example, in the intensity subgroup analysis, heterogeneity was concentrated in the high-intensity group (*I*^2^ = 75.7%). Additionally, when subgroups were stratified by outcome measures, studies that assessed muscle strength directly using instruments showed minimal heterogeneity, whereas studies using functional fitness tests exhibited very high within-group heterogeneity (*I*^2^ = 90.6%). These findings suggest that the observed high heterogeneity is the result of multiple contributing factors. First, higher-dose interventions tend to produce greater heterogeneity, likely because high-intensity or high-dose protocols are more sensitive to participants’ baseline fitness, skill proficiency, and individual physiological responses, thereby amplifying variability in intervention effects. Second, the type of outcome measure is an important source of heterogeneity. Direct instrument-based strength measurements have higher precision, whereas functional or mixed tests are influenced by multiple confounding factors, reflecting several dimensions of neuromuscular function (44), which ultimately leads to greater variability in results across studies.

The dose–response analysis provided statistically meaningful guidance for optimal exercise prescription. Cumulative effects were observed for exercise frequency, total weekly duration, and intervention period. Two to three sessions per week, totaling 100–150 min, produced stable strength gains, which aligns with ACSM recommendations that aquatic exercise should be performed at least twice weekly at moderate intensity (45). Other reviews have discussed weekly frequency: Bergamin et al. suggested three sessions per week as optimal (46), whereas Waller et al., based on a more comprehensive search and inclusion criteria, found no difference between two and three sessions (16). Our model predicted slightly better outcomes with three sessions (SMD = 0.62) compared to two sessions (SMD = 0.56), and the predicted effect increased robustly without a plateau. Regarding total weekly duration, gains plateaued after approximately 150–200 min, possibly due to older adults’ limited physiological reserves and recovery capacity. Sufficient recovery is necessary to avoid overtraining and ensure optimal adaptations (47), while exceeding a certain weekly time may reduce intrinsic motivation, despite the supportive aquatic environment (48). Moreover, longer intervention periods gradually increased effects up to 24 weeks, consistent with Deng et al. (49), indicating that sustained long-term engagement is essential for maximizing health benefits.

Single-session duration and exercise intensity exhibited an inverted U-shaped relationship. Model predictions indicated that a session of approximately 40 min at a moderate intensity (RPE 10–12) produced a highly effective response, while benefits decreased beyond this range.

Moderate exercise doses may enhance muscle strength in older adults through both physiological and psychological mechanisms. Insufficient intensity may fail to provide adequate physiological stimulus (50), whereas excessive doses can lead to diminishing returns. Prolonged high-intensity exercise may induce neuromuscular fatigue, reducing training quality and effective stimulus (51, 52). Additionally, excessive load can extend recovery time and undermine long-term adherence due to discomfort or frustration (48). In contrast, moderate exercise doses appear to balance muscle strengthening with fatigue risk. Recovery capacity is a key consideration in exercise prescription for older adults; meta-analyses by Cugusi et al. indicate that moderate-intensity aquatic exercise is also most effective in improving cardiovascular health indicators (53), and Hao Ying et al. (54) similarly concluded that moderate-intensity exercise is optimal for enhancing lower-limb muscle strength and function in older adults. These findings align with the present study, suggesting that an RPE of 10–12 represents a physiologically suitable intensity window for older adults.

The inverted U-shaped relationship highlights the importance of cautious exercise prescription in clinical and self-directed settings. Individual assessment and medical guidance are crucial to ensure that each older adult can benefit from exercise therapy while minimizing potential risks. Currently, there is a lack of clinical studies comparing different doses of aquatic exercise on physiological and psychological mechanisms in older adults, indicating a need for further high-quality trials to validate these conclusions.

## 5 Limitations

This study has several limitations. First, the literature search did not include gray literature, nor was expert consultation conducted to review the search strategy. The quality of the included randomized controlled trials varied, and potential risk of bias existed. Second, the quantification of various influencing parameters in the literature was not fully standardized. Although a standardized conversion procedure was implemented, unifying data from different measurement tools may still introduce errors and affect model fitting accuracy. Third, the variability in exercise types was not fully considered. Due to the small sample size, multiple outcome measures were combined in the effect size analysis to achieve sufficient statistical power, so the conclusions should be applied with caution. Fourth, this study primarily focused on healthy older adults and did not explore whether the dose–response relationships differ in populations with common chronic diseases, by sex, or among the “oldest-old” (≥75 years), In addition, we have noted in the limitations that some outcome measures may not fully capture daily functional capacity, and future studies should incorporate more comprehensive indicators of older adults’ functional ability. Therefore, extrapolation of the results to populations with specific comorbidities or different age groups should be approached cautiously. Finally, although the study examined dose–response relationships, it did not extensively elucidate the underlying pathophysiological and psychological mechanisms of the observed effects. Further research is needed to clarify the physiological and psychological mechanisms involved.

## 6 Conclusion and future directions

This study examined the effectiveness of aquatic exercise in improving muscle strength in older adults and, for the first time, systematically revealed the nonlinear dose–response relationships across different exercise dimensions. The findings aim to provide scientific support for exercise prescription and clinical practice for healthy older adults living in the community. Based on our model, it is recommended that healthy older adults engage in aquatic exercise two to three times per week, with sessions lasting approximately 40 min at a moderately high intensity (Borg RPE 10–12), as a long-term training strategy.

Future research should focus on conducting high-quality randomized controlled trials to compare the effects of the optimal dose identified in this study with other dosing regimens. Additionally, future research should place greater emphasis on precise recording and standardized reporting of exercise dose, to advance the development of more accurate and individualized aquatic exercise prescriptions for older adults.

## Data Availability

The original contributions presented in the study are included in the article/Supplementary material, further inquiries can be directed to the corresponding authors.

## Footnotes

1
www.r-project.org
