# Supplementary material for: Optimal dose of aquatic exercise for improving muscle strength in older adults: a Bayesian model-based meta-analysis
Source: Front Public Health. 2025 Dec 11;13:1699018. doi: 10.3389/fpubh.2025.1699018 (PMC12741266; doi:10.3389/fpubh.2025.1699018)
Supplement: Supplementary file 1 [file Table_1.docx]

**context**

[1. Supplementary 1: Search Strategy 3](#_Toc211521597)

[2. Supplementary 2: Characteristics of studies and subjects included in the review 6](#_Toc211521598)

[3. Supplementary 3: Risk of Bias 13](#_Toc211521599)

[4. Consistency 17](#_Toc211521600)

[5. Transitivity 18](#_Toc211521601)

[6. Non-linear functions and models fit comparison 32](#_Toc211521602)

[7. Models fit comparison 37](#_Toc211521603)

[8. deviance plots 41](#_Toc211521604)

[9. Model convergence degree 43](#_Toc211521605)

[10. Publication Bias 44](#_Toc211521606)

[11. PRISMA Checklist 46](#_Toc211521607)

[12. TIDieR scale analysis 54](#_Toc211521608)

[13. PEDro Scale Evaluation 55](#_Toc211521609)

# Supplementary 1: Search Strategy

**Database: PubMed <inception to March 30 2025> n= 601**

1. ("strength"[Title/Abstract] OR "muscle"[Title/Abstract] OR "muscle strength"[Title/Abstract] OR "muscular strength"[Title/Abstract] OR "neuromuscular function"[Title/Abstract] OR "neuromuscular response"[Title/Abstract])
2. #2 ("water exercise"[Title/Abstract] OR "aquatic exercise"[Title/Abstract] OR "water-based exercise"[Title/Abstract] OR "water aerobics"[Title/Abstract] OR "water aerobic exercise"[Title/Abstract] OR "aquatic resistance training"[Title/Abstract] OR "aquatic training"[Title/Abstract] OR "aquatic program"[Title/Abstract] OR "water fitness"[Title/Abstract] OR "aquatics"[Title/Abstract] OR "water training"[Title/Abstract] OR "deep water"[Title/Abstract] OR "water running"[Title/Abstract] OR "water walking"[Title/Abstract] OR "aquajogging"[Title/Abstract] OR "shallow water walking"[Title/Abstract] OR "hydrogymnastics"[Title/Abstract] OR "hydro"[Title/Abstract] OR "aquatic environment"[Title/Abstract] OR "water resistance"[Title/Abstract] OR "pool exercise"[Title/Abstract] OR hydrotherapy[Title/Abstract] OR balneotherapy[Title/Abstract])
3. #1 AND #2
4. (randomized controlled trial[pt] OR controlled clinical trial[pt] OR randomized[tiab] OR placebo[tiab] OR drug therapy[sh] OR randomly[tiab] OR trial[tiab] OR groups[tiab]) NOT (animals[mh] NOT humans[mh])
5. #3 AND #4

**Embase<inception to March 30 2025> n=246**

#1 ('strength'/exp OR 'muscle'/exp OR 'muscle strength'/exp OR 'muscular strength'/exp OR 'neuromuscular function'/exp OR 'neuromuscular response'/exp)

#2 ('water exercise'/exp OR 'aquatic exercise'/exp OR 'water-based exercise'/exp OR 'water aerobics'/exp OR 'water aerobic exercise'/exp OR 'aquatic resistance training'/exp OR 'aquatic training'/exp OR 'aquatic program'/exp OR 'water fitness'/exp OR 'aquatics'/exp OR 'water training'/exp OR 'deep water'/exp OR 'water running'/exp OR 'water walking'/exp OR 'aquajogging'/exp OR 'shallow water walking'/exp OR 'hydrogymnastics'/exp OR 'hydro'/exp OR 'aquatic environment'/exp OR 'water resistance'/exp OR 'pool exercise'/exp OR 'hydrotherapy'/exp OR 'balneotherapy'/exp)

#3 #1 AND #2

#4 ('randomized controlled trial'/exp)

#5 #3 AND #4

**Cochrane<inception to March 30 2025> n=367**

#1 ("strength":ti OR "muscle":ti OR "muscle strength":ti OR "muscular strength":ti OR "neuromuscular function":ti OR "neuromuscular response":ti OR "strength":ab OR "muscle":ab OR "muscle strength":ab OR "muscular strength":ab OR "neuromuscular function":ab OR "neuromuscular response":ab)

#2 ("water exercise":ti OR "aquatic exercise":ti OR "water-based exercise":ti OR "water aerobics":ti OR "water aerobic exercise":ti OR "aquatic resistance training":ti OR "aquatic training":ti OR "aquatic program":ti OR "water fitness":ti OR "aquatics":ti OR "water training":ti OR "deep water":ti OR "water running":ti OR "water walking":ti OR "aquajogging":ti OR "shallow water walking":ti OR "hydrogymnastics":ti OR "hydro":ti OR "aquatic environment":ti OR "water resistance":ti OR "pool exercise":ti OR hydrotherapy:ti OR balneotherapy:ti OR "water exercise":ab OR "aquatic exercise":ab OR "water-based exercise":ab OR "water aerobics":ab OR "water aerobic exercise":ab OR "aquatic resistance training":ab OR "aquatic training":ab OR "aquatic program":ab OR "water fitness":ab OR "aquatics":ab OR "water training":ab OR "deep water":ab OR "water running":ab OR "water walking":ab OR "aquajogging":ab OR "shallow water walking":ab OR "hydrogymnastics":ab OR "hydro":ab OR "aquatic environment":ab OR "water resistance":ab OR "pool exercise":ab OR hydrotherapy:ab OR balneotherapy:ab)

#3 #1 AND #2

#4 ("randomized controlled trial":pt OR "controlled clinical trial":pt OR "randomized":tiab OR "placebo":tiab OR "drug therapy":sh OR "randomly":tiab OR "trial":tiab OR "groups":tiab) NOT (animals[mh] NOT humans[mh])

#5 #3 AND #4

**WEB OF SCIENCE<inception to March 30 2025> n=379**

#1 TS=("strength" OR "muscle" OR "muscle strength" OR "muscular strength" OR "neuromuscular function" OR "neuromuscular response")

#2 TS=("water exercise" OR "aquatic exercise" OR "water-based exercise" OR "water aerobics" OR "water aerobic exercise" OR "aquatic resistance training" OR "aquatic training" OR "aquatic program" OR "water fitness" OR "water fitness program" OR "aquatics" OR "water resistance training" OR "water training" OR "deep water training" OR "deep water" OR "water running" OR "water walking" OR "aquajogging" OR "shallow water walking" OR "hydrogymnastics" OR "hydro" OR "aqua gym" OR "aquatic environment" OR "water resistance" OR "pool exercise" OR hydrotherapy OR balneotherapy)

#3 #1 AND #2

#4 TS=("randomized controlled trial" OR "controlled clinical trial" OR "randomized" OR "randomly" OR "placebo" OR "clinical trial" OR "controlled trial" OR "double-blind" OR "single-blind")

#5 #3 AND #4

**CNKI<inception to March 30 2025> n=367**

#1 题名或关键词 肌肉力量 + 肌力 + 肌肉强度 + 肌肉功能 + 神经肌肉功能 + 神经肌肉反应

#2 题名或关键词 水中运动 + 水疗 + 水中训练 + 水中健身 + 水中有氧运动 + 水中阻力训练 + 水池训练 + 水阻力 + 水中训练

#3 #1 AND #2

#4 (TI='随机对照试验' OR TI='随机临床试验' OR TI='随机' OR TI='安慰剂' OR TI='随机分组' OR TI='试验' OR TI='分组') NOT SU='动物'

#5 #3 AND #4

**Wan Fang<inception to March 30 2025> n=120**

#1 肌肉力量 OR 肌力 OR 肌肉强度 OR 肌肉功能 OR 神经肌肉功能 OR 神经肌肉反应

#2 水中运动 OR 水疗 OR 水中训练 OR 水中健身 OR 水中有氧运动 OR 水中阻力训练 OR 水池训练 OR 水阻力 OR 水中训练

#3 #1 AND #2

#4 随机对照试验 OR 随机临床试验 OR 随机 OR 安慰剂 OR 随机分组 OR 试验 OR 分组 NOT 动物

#5 #3 AND #4

Table 1 Number of records retrieved from each database and corresponding search dates

| Database | Number of Records Identified | Date of Search |
| --- | --- | --- |
| PubMed | 601 | March 30, 2025 |
| Embase | 246 | March 30, 2025 |
| Cochrane | 367 | March 30, 2025 |
| Web of Science | 379 | March 30, 2025 |
| CNKI | 367 | March 30, 2025 |
| Wang Fang | 120 | March 30, 2025 |

# Supplementary 2: Characteristics of studies and subjects included in the review

| Number | Study | Country | Participants | Age (mean ± sd) | Sample Size (F/M) & Groups | Intervention Protocol | Feature of Study |
| --- | --- | --- | --- | --- | --- | --- | --- |
| 1 | Bergamin et al. (2013) | Italy | Healthy older adults | >65 years (71.2 ± 5.4) | Aquatic Group (AG): 20; Control Group (CG): 19 | Duration: 24 weeks; Frequency: 2 times/week; Session: 60 min; Content: Program included 8-min warm-up, upper body exercises, and 8-min cool-down. | Water depth: 1.3 – 1.8m  Water temperature:  36.2°C  Resistance Equipment: no |
| 2 | Bocalini et al. (2008) | Brazil | Older adults | WE: 64±1; WL: 64±1 | Water Exercise (WE): 27; Land-based (WL): 25 | Duration: 12 weeks; Frequency: 3 times/week; Session: 60 min; Content: WE group: 10-min warm-up, 45-min endurance exercise, 5-min cool-down. WL group: 30-min walking. | Water depth: N/A  Water temperature:  N/A  Resistance Equipment: yes |
| 3 | Bento et al. (2012) | Brazil | Older adults | WBG: 65.6±4.2; CG: 65.6±4.4 | Water-based Group (WBG): 24; Control Group (CG): 14 | Duration: 12 weeks; Frequency: 3 times/week; Session: 60 min; Content: 10-min warm-up, 20-min aerobics, 20-min lower limb strength training, 10-min stretching. | Water depth: N/A  Water temperature:  N/A  Resistance Equipment: yes (specialized water-resistance equipment for upper and lower body) |
| 4 | Chen et al. (2024) | China | Older adults | 60–70 years (66.2 ± 2.3) | Aquatic Exercise Group (FS): 20; Control Group: 20 | Duration: 16 weeks; Frequency: 2 times/week; Session: 60 min; Content: 5-min upper body/trunk + 5-min lower limb warm-up; FS group performed HIIT; ART group performed limb & trunk resistance training. | Water depth: N/A  Water temperature:  N/A  Resistance Equipment: no |
| 5 | Ferreira et al. (2022) | Brazil | Community-dwelling older adults | ATG: 70.15±4.24; CG: 71.40±4.57 | Aquatic Training Group (ATG): 24; Control Group (CG): 25 | Duration: 16 weeks; Frequency: 2 times/week; Session: 60 min; Content: Multi-component aquatic training (20-min resistance, 15-min balance, 10-min aerobic) combined with cognitive tasks. | Water depth: N/A  Water temperature:  N/A  Resistance Equipment: no |
| 6 | Alejandro et al. (2022) | Spain | Healthy older women (>65) | EG: 69.4 ± 4.9; CG: 67.7 ± 3.6 | Experimental Group (EG): 17 (F); Control Group (CG): 17 (F) | Duration: 14 weeks; Frequency: 3 times/week; Session: 60 min; Content: Aquatic resistance interval training (15-min warm-up, 4 sets × 5-min main training, 2-min rest between sets). | Water depth: N/A  Water temperature:  N/A  Resistance Equipment: no |
| 7 | Moreira et al. (2020) | Brazil | Older adults with a history of falls | AG: 70.6±6.0; CG: 71.9±6.9 | Aquatic Group (AG): 60 (49F/11M); Control Group (CG): 60 (43F/17M) | Duration: 16 weeks; Frequency: 2 times/week; Session: 45 min; Content: Progressive aquatic resistance training at moderate intensity (Borg 12–16). | Water depth: N/A  Water temperature:  N/A  Resistance Equipment: no |
| 8 | Oh et al. (2015) | South Korea | Community-dwelling older adults with a history of falls | WE: 74.71±2.9; CG: 68.21±4.4 | Water Exercise (WE): 34; Land-based Group: 32 | Duration: 10 weeks; Frequency: 3 times/week; Session: 60 min; Content: Strength and endurance exercises, including multi-directional resistance activities for shoulders and hips while single-leg standing. | Water depth: N/A  Water temperature:  N/A  Resistance Equipment: no |
| 9 | Takeshima et al. (2002) | Japan | Older women | WE: 69.3±4.5; CG: 69.3±3.3 | Water Exercise (WE): 15 (F); Control Group (CG): 15 (F) | Duration: 12 weeks; Frequency: 3 times/week; Session: 70 min; Content: Multi-component training (20-min warm-up/stretching, 30-min aerobic, 10-min resistance, 10-min cool-down). | Water depth: near xiphoid level  Water temperature:  30°C  Resistance Equipment: yes (Finbell water-resistance products: hand bars, leg pads) |
| 10 | Taunton et al. (1996) | Canada | Older women | 65–75 years (70±3.2) | Water Exercise (WE): 18 (F); Control Group (CG): 13 (F) | Duration: 12 weeks; Frequency: 3 times/week; Session: 45 min; Content: Aquatic exercise with intensity set at 60–65% of maximum heart rate. | Water depth: N/A  Water temperature:  N/A  Resistance Equipment: no |
| 11 | Tsourlou et al. (2006) | Greece | Healthy older women | WE: 69.3±1.9; CG: 68.4±6.7 | Water Exercise (WE): 12 (F); Control Group (CG): 10 (F) | Duration: 24 weeks; Frequency: 3 times/week; Session: 60 min; Content: 10-min warm-up, 25-min aerobic dance (65–80% MHR), 20–25 min resistance training (using aquatic equipment), 5-min cool-down. | Water depth: N/A  Water temperature:  N/A  Resistance Equipment: yes |
| 12 | Graef et al. (2010) | Brazil | Brazilian older women | RWE: 68.4±6.7; WE: 64.1±3.5; CG: 67.6±4.7 | RWE: 10 (F); WE: 10 (F); CG: 7 (F) | Duration: 12 weeks; Frequency: 2 times/week; Session: 50 min; Content: RWE group performed aquatic resistance training at maximal velocity (using foam paddles); WE group performed non-resistance aquatic exercises. | Water depth: N/A  Water temperature:  N/A  Resistance Equipment: yes (specialized water-resistance equipment for upper/lower body)  (resistive equipment for shoulder exercises) |
| 13 | Kim et al. (2013) | South Korea | Korean older women | WE: 70.86±4.97; CG: 72.57±5.09 | Water Exercise (WE): 8 (F); Control Group (CG): 7 (F) | Duration: 12 weeks; Frequency: 3 times/week; Session: 60 min; Content: 10-min warm-up, 10-min adaptation, 30-min main exercises (shallow water walking, squats, dumbbells; deep water coordination & balance), 10-min cool-down. | Water depth: 1.3 m  Water temperature:  28±1°C  Resistance Equipment: yes (dumbbells, boxes, noodles, balls for muscle stimuli) |

# Supplementary 3: Risk of Bias

| Number | Study | Random sequence generation | Allocation concealment | Blinding of outcome assessment | Incomplete outcome data | Selective reporting | Other bias | Overall judgement |
| --- | --- | --- | --- | --- | --- | --- | --- | --- |
| 1 | Bergamin et al. (2013) | Low | Unclear | High | Low | Unclear | Unclear | High |
| 2 | Bocalini et al. (2008) | Low | Unclear | High | Low | Low | Unclear | High |
| 3 | Bento et al. (2012) | Low | Unclear | High | Low | Low | Unclear | High |
| 4 | Chen et al. (2024) | Low | Unclear | Low | Low | Low | Unclear | Unclear |
| 5 | Ferreira et al. (2022) | Low | Low | High | High | High | Unclear | High |
| 6 | Alejandro et al. (2022) | Low | Unclear | Low | Low | Low | Unclear | Unclear |
| 7 | Moreira et al. (2020) | Low | Unclear | High | Low | Low | Unclear | High |
| 8 | Oh et al. (2015) | Low | Low | Low | Low | Unclear | Unclear | Unclear |
| 9 | Takeshima et al. (2002) | Low | Unclear | High | Low | Unclear | Unclear | Unclear |
| 10 | Taunton et al. (1996) | Low | Unclear | High | Low | Low | Unclear | High |
| 11 | Tsourlou et al. (2006) | Low | Unclear | High | Low | Low | Unclear | High |
| 12 | Graef et al. (2010) | Low | Low | High | Low | Unclear | Unclear | High |
| 13 | Kim et al. (2013) | Low | Low | Low | Low | Low | Unclear | Unclear |

Overall judgement was determined according to the Cochrane Handbook (version 5.1.0, Chapter 8): studies were rated as “Low” if all domains were at low risk of bias; “High” if one or more domains were at high risk; and “Unclear” if one or more domains were judged unclear and none were high risk.

Methodological quality was additionally evaluated using the PEDro scale, and scores are provided in the supplementary table.

Table 3-1 Inter-rater agreement for risk of bias assessment across included studies (Cohen’s Kappa values)

| **Dimension** | **Kappa** | **Remark** |
| --- | --- | --- |
| Random sequence generation | NA | Cannot compute (single category) |
| Allocation concealment | 1.000 | Almost perfect agreement |
| Blinding of outcome assessment | 0.831 | Almost perfect agreement |
| Incomplete outcome data | 1.000 | Almost perfect agreement |
| Selective reporting | 0.690 | Substantial agreement |
| Other bias | NA | Cannot compute (single category) |

Table 3-1 presents the inter-rater agreement between two reviewers for each risk of bias domain across the included studies, expressed as Cohen’s Kappa values.

The Kappa values for “Allocation concealment” and “Incomplete outcome data” are both 1.000, indicating perfect agreement between the two reviewers.

The Kappa value for “Blinding of outcome assessment” is 0.831, indicating almost perfect agreement.

The Kappa value for “Selective reporting” is 0.690, indicating substantial agreement.

The Kappa values for “Random sequence generation” and “Other bias” could not be calculated (shown as NaN) because both reviewers assigned a single category for these domains, resulting in no category variation. This does not indicate disagreement; rather, it shows complete agreement, and Kappa cannot be computed in such cases.

List of included studies

1. Bento P C B, Pereira G, Ugrinowitsch C, et al. The effects of a water-based exercise program on strength and functionality of older adults[J]. Journal of aging and physical activity, 2012, 20(4): 469-470.
2. Bergamin M, Ermolao A, Tolomio S, et al. Water-versus land-based exercise in elderly subjects: effects on physical performance and body composition[J]. Clinical interventions in aging, 2013: 1109-1117.
3. Bocalini D S, Serra A J, Murad N, et al. Water‐versus land‐based exercise effects on physical fitness in older women[J]. Geriatrics & gerontology international, 2008, 8(4): 265-271.
4. Chen Y, Lan Y, Zhao A H, et al. High-intensity interval swimming improves cardiovascular endurance, while aquatic resistance training enhances muscular strength in older adults[J]. Scientific reports, 2024, 14(1): 25241.
5. Ferreira D L, Christofoletti G, Campos D M, et al. Effects of aquatic physical exercise on motor risk factors for falls in older people during the COVID-19 pandemic: a randomized controlled trial[J]. Journal of manipulative and physiological therapeutics, 2022, 45(5): 378-388.
6. Martínez-Rodríguez A, Cuestas-Calero B J, García de Frutos J M, et al. Effect of aquatic resistance interval training and dietary education program on physical and psychological health in older women: Randomized controlled trial[J]. Frontiers in Nutrition, 2022, 9: 980788.
7. Moreira N B, da Silva L P, Rodacki A L F. Aquatic exercise improves functional capacity, perceptual aspects, and quality of life in older adults with musculoskeletal disorders and risk of falling: A randomized controlled trial[J]. Experimental gerontology, 2020, 142: 111135.
8. Oh S J, Lim J M, Kim Y, et al. Comparison of the effects of water-and land-based exercises on the physical function and quality of life in community-dwelling elderly people with history of falling: a single-blind, randomized controlled trial[J]. Archives of Gerontology and Geriatrics, 2015, 60(2): 288-293.
9. Takeshima N, Rogers M E, Watanabe E, et al. Water-based exercise improves health-related aspects of fitness in older women[J]. Medicine & Science in Sports & Exercise, 2002, 34(3): 544-551.
10. Taunton J E, Rhodes E C, Wolski L A, et al. Effect of land-based and water-based fitness programs on the cardiovascular fitness, strength and flexibility of women aged 65–75 years[J]. Gerontology, 1996, 42(4): 204-210.
11. Tsourlou T, Benik A, Dipla K, et al. The effects of a twenty-four--week aquatic training program on muscular strength performance in healthy elderly women[J]. The Journal of Strength & Conditioning Research, 2006, 20(4): 811-818.
12. Graef F I, Pinto R S, Alberton C L, et al. The effects of resistance training performed in water on muscle strength in the elderly[J]. The Journal of Strength & Conditioning Research, 2010, 24(11): 3150-3156.
13. Kim S B, O’sullivan D M. Effects of aqua aerobic therapy exercise for older adults on muscular strength, agility and balance to prevent falling during gait[J]. Journal of physical therapy science, 2013, 25(8): 923-927

# Consistency

Table 2 Consistent and UME models fit comparison

| Variable | Model | pD | Residual deviance | Deviance | DIC | SD |
| --- | --- | --- | --- | --- | --- | --- |
| period | Consistent | 45.8 | 42.634 | 125.451 | 171.2 | 68.495 |
| period | UME | 40.3 | 43.741 | 126.558 | 167.3 | 63.169 |
| frequency | Consistent | 44.5 | 43.406 | 126.223 | 170.7 | 64.615 |
| frequency | UME | 40.1 | 43.606 | 126.422 | 166.7 | 63.056 |
| time | Consistent | 45.9 | 43.173 | 125.99 | 171.9 | 67.51 |
| time | UME | 40.2 | 43.774 | 126.591 | 167.0 | 63.364 |
| weekly time | Consistent | 46.4 | 42.943 | 125.76 | 172.1 | 70.081 |
| weekly time | UME | 39.5 | 43.766 | 126.583 | 165.9 | 63.074 |
| intensity | Consistent | 30.6 | 30.579 | 89.216 | 119.8 | 39.264 |
| intensity | UME | 30.1 | 31.46 | 90.097 | 120.0 | 32.281 |

# Transitivity

## Period

Table 3 Node-splitting analysis of inconsistency

| Comparison | p-value | Median | 2.5% | 97.5% |
| --- | --- | --- | --- | --- |
| AE_24 vs Placebo_0 | 0.313 |  |  |  |
| → direct |  | 7.604 | 1.699 | 13.523 |
| → indirect |  | -2.758 | -28.584 | 22.617 |
| → MBNMA |  | 7.124 | 1.726 | 12.561 |
|  |  |  |  |  |
| AE_16 vs Placebo_0 | 0.396 |  |  |  |
| → direct |  | 1.221 | -4.009 | 6.199 |
| → indirect |  | 4.728 | 1.187 | 8.931 |
| → MBNMA |  | 3.437 | 0.652 | 6.624 |
|  |  |  |  |  |
| AE_14 vs Placebo_0 | 0.208 |  |  |  |
| → direct |  | 10.272 | -0.449 | 23.186 |
| → indirect |  | 2.014 | -1.048 | 5.504 |
| → MBNMA |  | 2.699 | -0.278 | 6.016 |
|  |  |  |  |  |
| AE_12 vs Placebo_0 | 0.844 |  |  |  |
| → direct |  | 2.509 | -2.036 | 7.714 |
| → indirect |  | 1.555 | -3.404 | 6.934 |
| → MBNMA |  | 2.016 | -1.120 | 5.495 |
|  |  |  |  |  |
| AE_10 vs Placebo_0 | 0.480 |  |  |  |
| → direct |  | 1.562 | -10.537 | 13.471 |
| → indirect |  | 1.542 | -1.861 | 5.505 |
| → MBNMA |  | 1.495 | -1.566 | 4.953 |


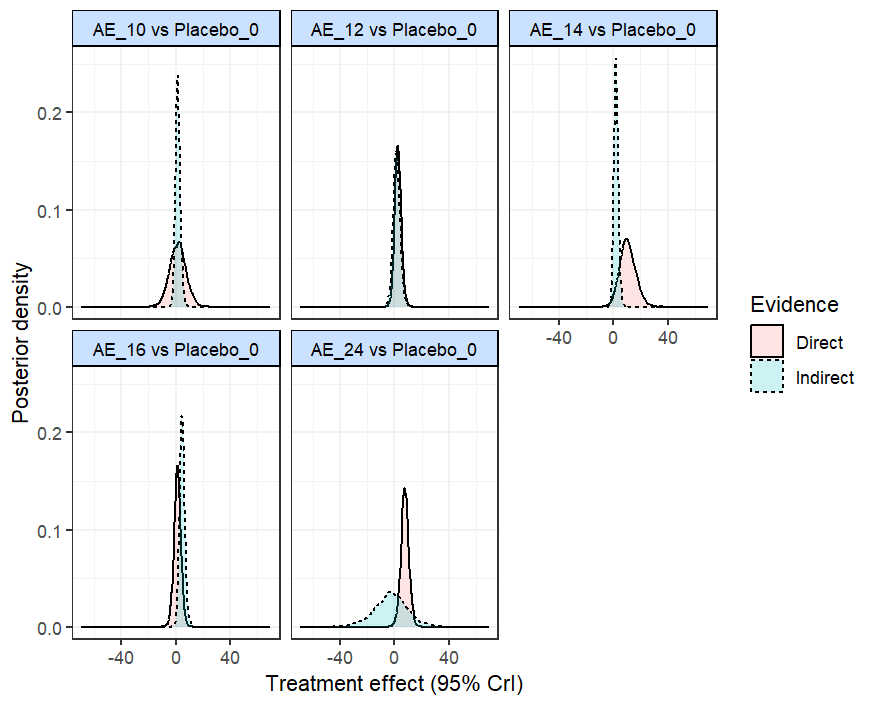


Figure 1 Node-splitting analysis (density plot). The first value indicates the agent and the second one is the corresponding dose of that agent. *AE* Aquatic Exercise

## Frequency

Table 4 Node-splitting analysis of inconsistency

| Comparison | p-value | Median | 2.5% | 97.5% |
| --- | --- | --- | --- | --- |
| AE_3 vs Placebo_0 | 0.066 |  |  |  |
| → direct |  | 5.858 | 2.066 | 10.156 |
| → indirect |  | -0.813 | -145.270 | 150.282 |
| → MBNMA |  | 5.791 | 2.031 | 9.991 |
|  |  |  |  |  |
| AE_2 vs Placebo_0 | 0.097 |  |  |  |
| → direct |  | 1.444 | -2.817 | 5.569 |
| → indirect |  | 0.278 | -99.644 | 99.364 |
| → MBNMA |  | 1.308 | -2.905 | 5.400 |


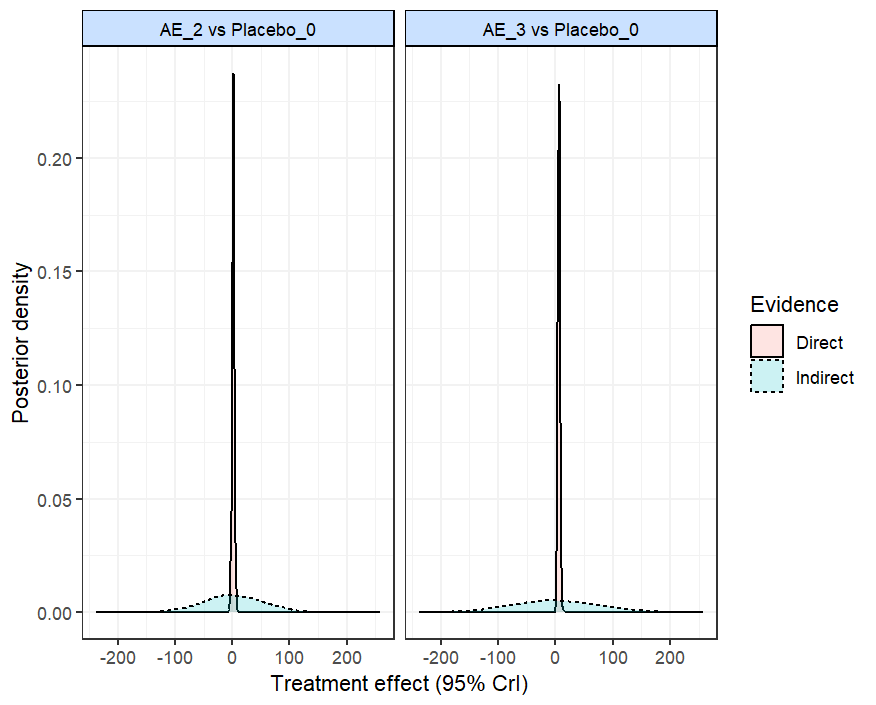


Figure 2 Node-splitting analysis (density plot). The first value indicates the agent and the second one is the corresponding dose of that agent. *AE* Aquatic Exercise

## Time

Table 5 Node-splitting analysis of inconsistency

| Comparison | p-value | Median | 2.5% | 97.5% |
| --- | --- | --- | --- | --- |
| AE_70 vs Placebo_0 | 0.811 |  |  |  |
| → direct |  | 6.603 | -5.019 | 19.441 |
| → indirect |  | 4.380 | -4.900 | 14.752 |
| → MBNMA |  | 5.314 | -1.609 | 12.785 |
|  |  |  |  |  |
| AE_60 vs Placebo_0 | 0.680 |  |  |  |
| → direct |  | 3.844 | 0.215 | 7.731 |
| → indirect |  | 4.946 | -1.545 | 12.104 |
| → MBNMA |  | 4.064 | 1.118 | 7.408 |
|  |  |  |  |  |
| AE_50 vs Placebo_0 | 0.747 |  |  |  |
| → direct |  | 3.512 | -6.395 | 12.865 |
| → indirect |  | 2.670 | -3.107 | 8.607 |
| → MBNMA |  | 2.946 | -1.941 | 7.714 |
|  |  |  |  |  |
| AE_45 vs Placebo_0 | 0.926 |  |  |  |
| → direct |  | 2.655 | -6.618 | 11.540 |
| → indirect |  | 2.042 | -8.208 | 12.146 |
| → MBNMA |  | 2.446 | -4.022 | 8.716 |


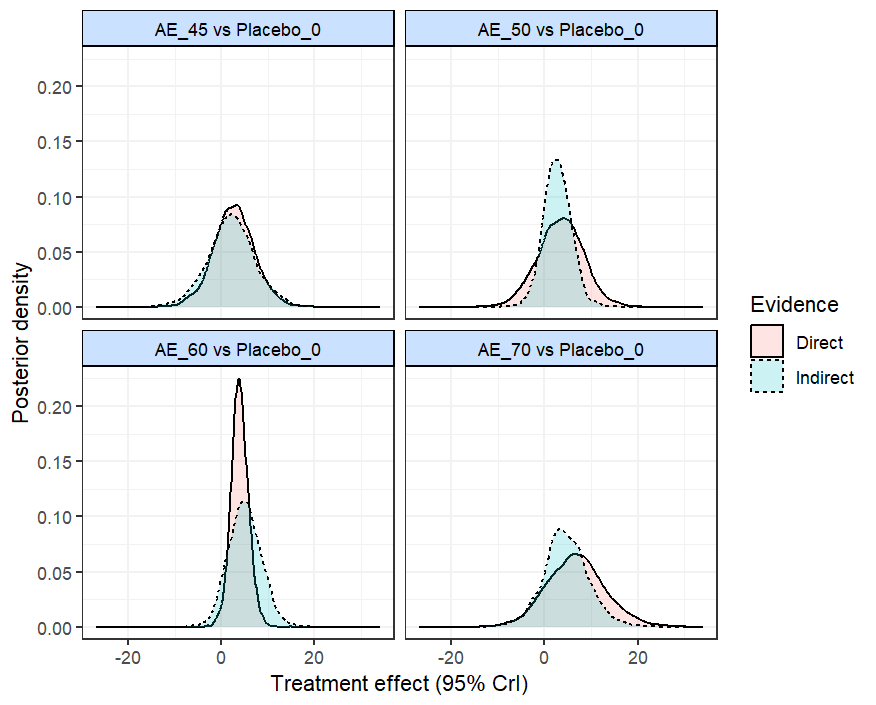


Figure 3 Node-splitting analysis (density plot). The first value indicates the agent and the second one is the corresponding dose of that agent. AE Aquatic Exercise

## Weekly time

Table 6 Node-splitting analysis of inconsistency

| Comparison | p-value | Median | 2.5% | 97.5% |
| --- | --- | --- | --- | --- |
| AE_210 vs Placebo_0 | 0.757 |  |  |  |
| → direct |  | 6.451 | -4.037 | 18.643 |
| → indirect |  | 8.227 | 0.921 | 16.332 |
| → MBNMA |  | 7.584 | 1.441 | 14.019 |
|  |  |  |  |  |
| AE_180 vs Placebo_0 | 0.708 |  |  |  |
| → direct |  | 5.898 | 1.897 | 10.838 |
| → indirect |  | 4.315 | -2.568 | 11.500 |
| → MBNMA |  | 5.485 | 1.725 | 9.309 |
|  |  |  |  |  |
| AE_150 vs Placebo_0 | 0.419 |  |  |  |
| → direct |  | 4.147 | -9.804 | 17.693 |
| → indirect |  | 3.578 | 0.341 | 6.985 |
| → MBNMA |  | 3.512 | 0.468 | 6.722 |
|  |  |  |  |  |
| AE_120 vs Placebo_0 | 0.573 |  |  |  |
| → direct |  | 0.589 | -4.871 | 5.989 |
| → indirect |  | 3.560 | -2.369 | 9.665 |
| → MBNMA |  | 1.927 | -2.126 | 5.954 |
|  |  |  |  |  |
| AE_100 vs Placebo_0 | 0.548 |  |  |  |
| → direct |  | 2.605 | -9.841 | 15.178 |
| → indirect |  | 0.991 | -3.801 | 5.859 |
| → MBNMA |  | 1.166 | -3.206 | 5.504 |
|  |  |  |  |  |
| AE_90 vs Placebo_0 | 0.645 |  |  |  |
| → direct |  | 2.537 | -6.369 | 11.047 |
| → indirect |  | 0.186 | -4.773 | 5.652 |
| → MBNMA |  | 0.866 | -3.504 | 5.212 |


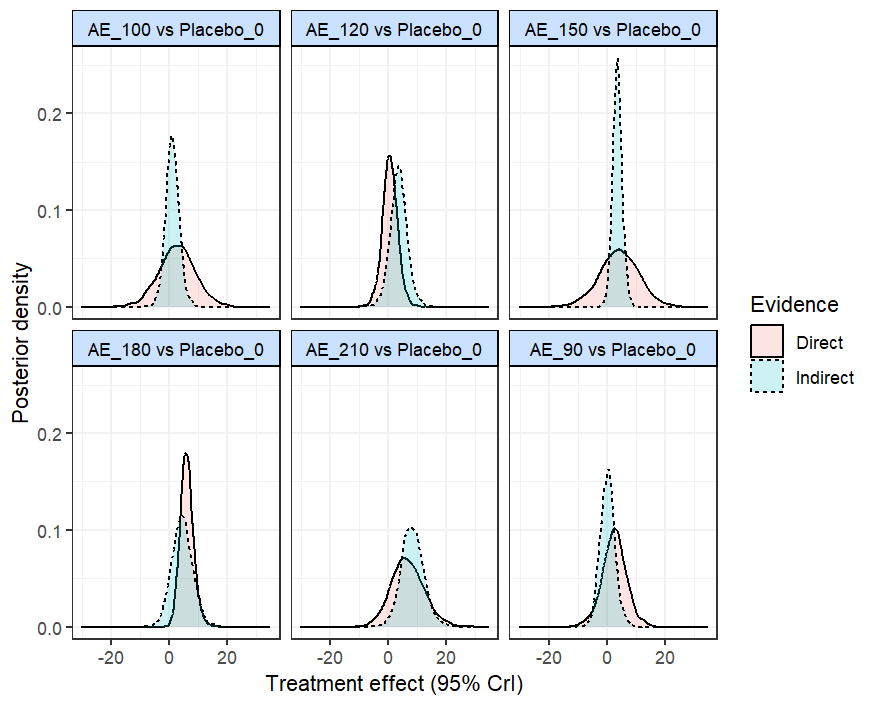


Figure 4 Node-splitting analysis (density plot). The first value indicates the agent and the second one is the corresponding dose of that agent. AE Aquatic Exercise

## Intensity

Table 7 Node-splitting analysis of inconsistency

| Comparison | p-value | Median | 2.5% | 97.5% |
| --- | --- | --- | --- | --- |
| AE_210 vs Placebo_0 | 0.766 |  |  |  |
| → direct |  | 6.461 | -5.140 | 18.935 |
| → indirect |  | 8.194 | 0.412 | 16.792 |
| → MBNMA |  | 7.494 | 1.860 | 14.270 |
|  |  |  |  |  |
| AE_180 vs Placebo_0 | 0.709 |  |  |  |
| → direct |  | 6.084 | 1.542 | 10.958 |
| → indirect |  | 4.429 | -2.630 | 12.106 |
| → MBNMA |  | 5.471 | 2.030 | 9.492 |
|  |  |  |  |  |
| AE_150 vs Placebo_0 | 0.424 |  |  |  |
| → direct |  | 4.039 | -9.153 | 17.328 |
| → indirect |  | 3.522 | 0.480 | 7.270 |
| → MBNMA |  | 3.557 | 0.754 | 6.821 |
|  |  |  |  |  |
| AE_120 vs Placebo_0 | 0.588 |  |  |  |
| → direct |  | 0.494 | -5.061 | 6.332 |
| → indirect |  | 3.587 | -2.359 | 9.444 |
| → MBNMA |  | 1.964 | -1.753 | 5.847 |
|  |  |  |  |  |
| AE_100 vs Placebo_0 | 0.565 |  |  |  |
| → direct |  | 2.511 | -9.877 | 15.188 |
| → indirect |  | 0.963 | -3.617 | 5.601 |
| → MBNMA |  | 1.183 | -3.080 | 5.318 |
|  |  |  |  |  |
| AE_90 vs Placebo_0 | 0.651 |  |  |  |
| → direct |  | 2.630 | -5.934 | 10.985 |
| → indirect |  | 0.213 | -5.083 | 5.305 |
| → MBNMA |  | 0.899 | -3.399 | 4.942 |


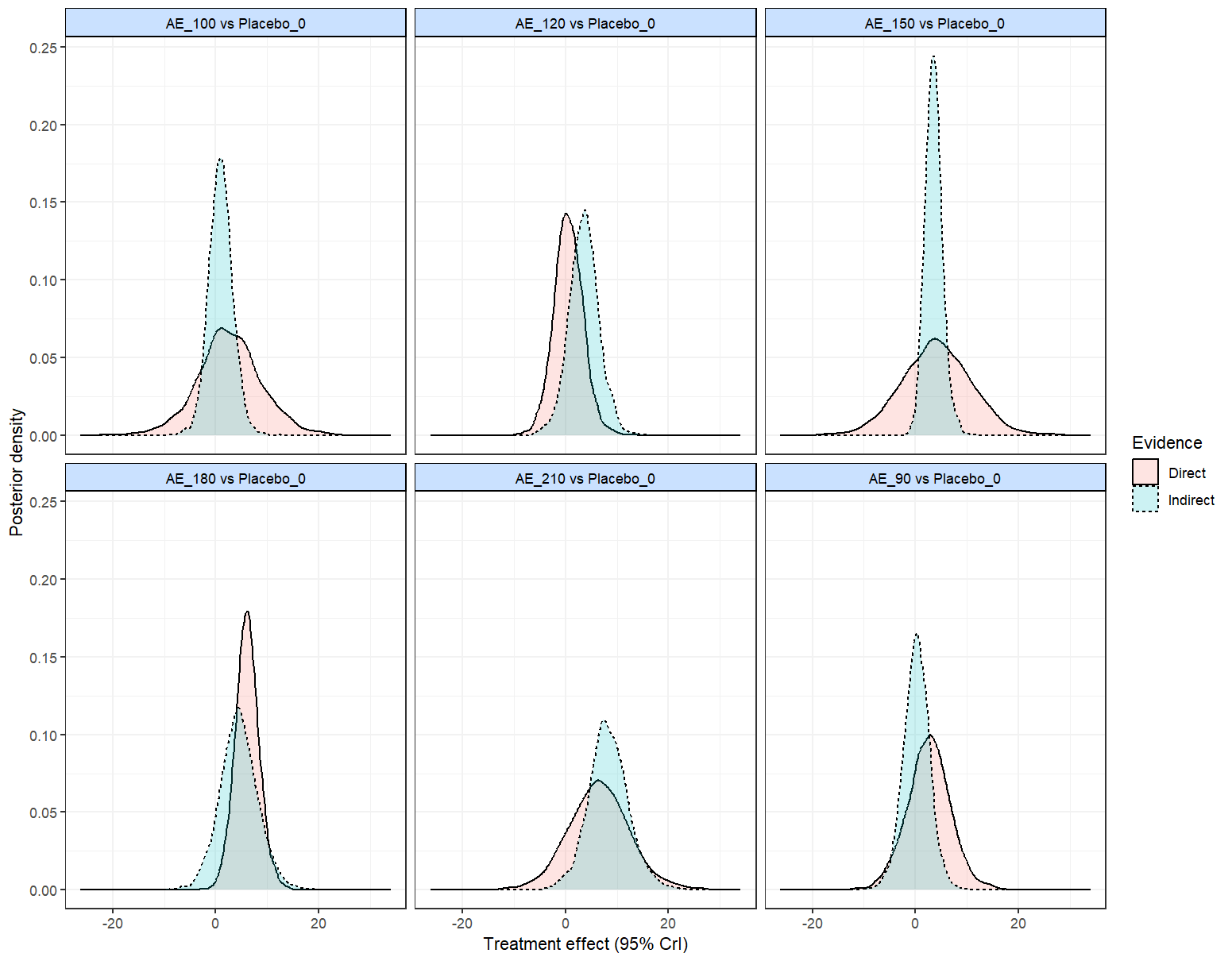


Figure 5 Node-splitting analysis (density plot). The first value indicates the agent and the second one is the corresponding dose of that agent. AE Aquatic Exercise

# Non-linear functions and models fit comparison

## Period


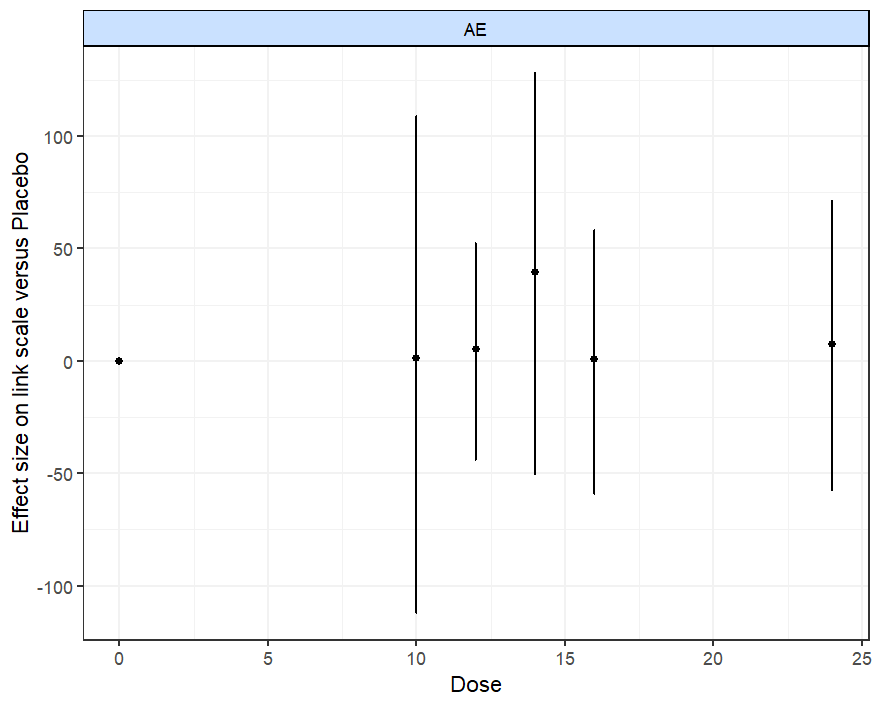


Figure 6 “Split” NMA of Aquatic exercise agents. *AE* Aquatic Exercise.

## Frequency


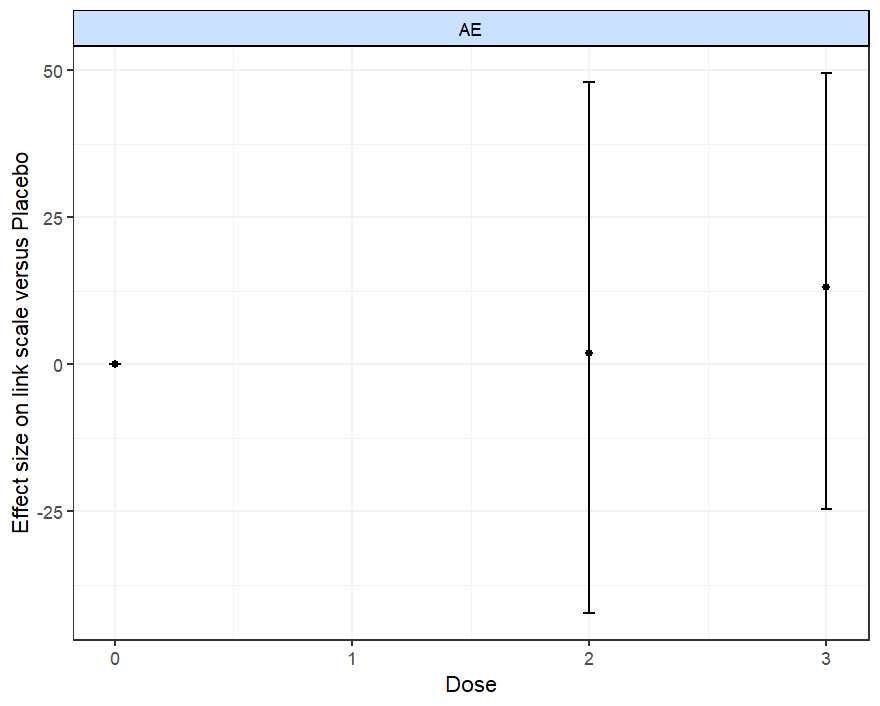


Figure 7 “Split” NMA of Aquatic exercise agents. AE Aquatic Exercise.

## Time


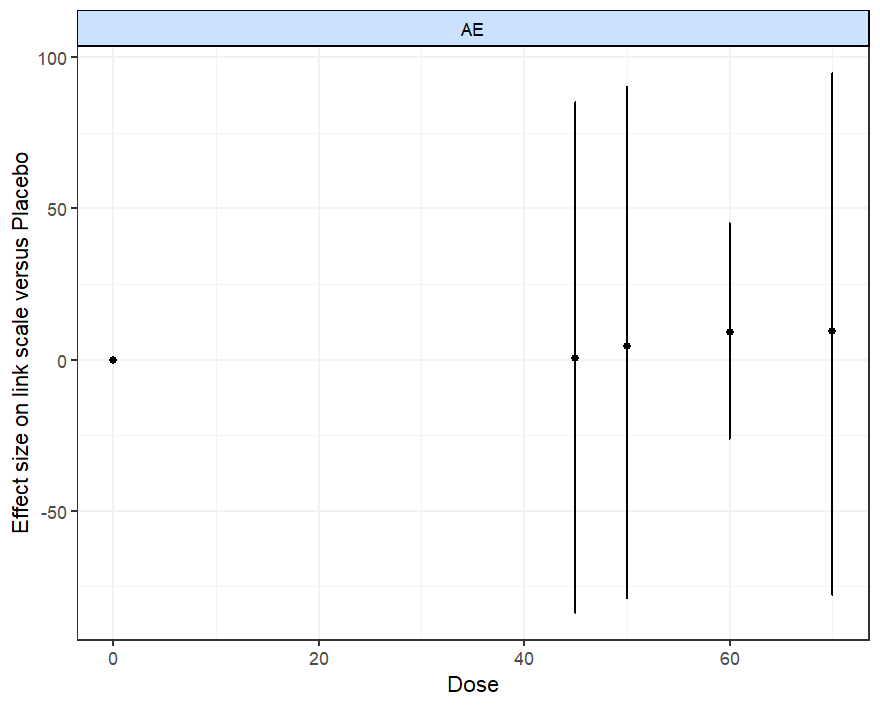


Figure 8 “Split” NMA of Aquatic exercise agents. AE Aquatic Exercise.

## Weekly time


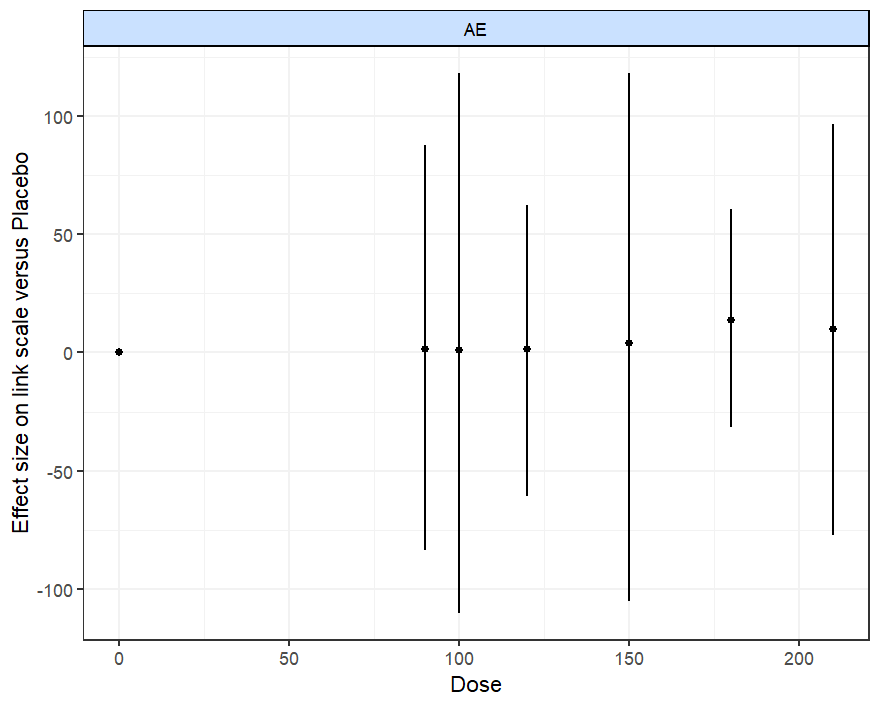


Figure 9 “Split” NMA of Aquatic exercise agents. AE Aquatic Exercise.

## Intensity


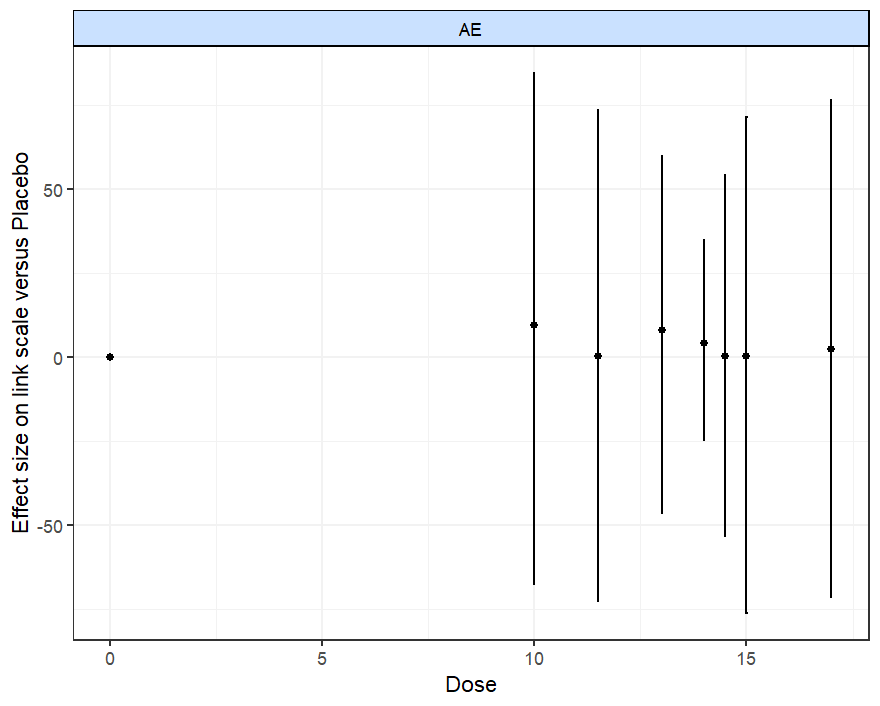


Figure 10 “Split” NMA of Aquatic exercise agents. AE Aquatic Exercise.

# Models fit comparison

## Period

| MODEL | DIC | SD | DEVIANCE | RESIDUAL DEVIANCE | pD |
| --- | --- | --- | --- | --- | --- |
| EMAX (COMMON TREATMENT EFFECTS) | 8853.8 | - | 8839.055 | 8756.238 | 14.3 |
| EMAX (RANDOM TREATMENT EFFECTS) | 166.2 | 62.403 | 126.791 | 43.974 | 39.8 |
| LINEAR (COMMON TREATMENT EFFECTS) | 8925.4 | - | 8911.155 | 8828.338 | 14.1 |
| LINEAR (RANDOM TREATMENT EFFECTS) | 162.1 | 5.574 | 122.897 | 40.080 | 39.7 |
| EXPONENTIAL (COMMON TREATMENT EFFECTS) | 8854.5 | - | 8841.355 | 8758.538 | 14.8 |
| EXPONENTIAL (RANDOM TREATMENT EFFECTS) | 165.8 | 62.876 | 126.376 | 43.560 | 39.6 |
| RESTRICTED CUBIC SPLINE (COMMON TREATMENT EFFECTS; 3 KNOTS) | 8925.4 | - | 8910.483 | 8827.666 | 14.9 |
| RESTRICTED CUBIC SPLINE (RANDOM TREATMENT EFFECTS; 3 KNOTS) | 161.6 | 5.713 | 122.911 | 40.094 | 39.5 |
| NON-PARAMETRIC MONOTONICALLY UP (COMMON TREATMENT EFFECTS) | 8853.6 | - | 8838.288 | 8755.471 | 15.5 |
| NON-PARAMETRIC MONOTONICALLY UP (RANDOM TREATMENT EFFECTS) | 165.5 | 69.908 | 125.159 | 42.343 | 40.0 |

## Frequency

| MODEL | DIC | SD | DEVIANCE | RESIDUAL DEVIANCE | pD |
| --- | --- | --- | --- | --- | --- |
| EMAX (COMMON TREATMENT EFFECTS) | 8868.6 | – | 8854.727 | 8771.910 | 14.0 |
| EMAX (RANDOM TREATMENT EFFECTS) | 165.5 | 61.964 | 126.611 | 43.794 | 39.3 |
| LINEAR (COMMON TREATMENT EFFECTS) | 8921.4 | – | 8907.737 | 8824.920 | 13.8 |
| LINEAR (RANDOM TREATMENT EFFECTS) | 161.5 | 5.598 | 122.463 | 39.646 | 39.2 |
| EXPONENTIAL (COMMON TREATMENT EFFECTS) | 8870.4 | – | 8856.159 | 8773.342 | 14.5 |
| EXPONENTIAL (RANDOM TREATMENT EFFECTS) | 167.1 | 63.521 | 126.673 | 43.856 | 40.2 |
| RESTRICTED CUBIC SPLINE (COMMON TREATMENT EFFECTS; 3 KNOTS) | 8923.9 | – | 8908.545 | 8825.729 | 15.1 |
| RESTRICTED CUBIC SPLINE (RANDOM TREATMENT EFFECTS; 3 KNOTS) | 162.5 | 5.687 | 122.857 | 40.040 | 39.7 |
| NON-PARAMETRIC MONOTONICALLY UP (COMMON TREATMENT EFFECTS) | 8869.9 | – | 8855.194 | 8772.377 | 14.6 |
| NON-PARAMETRIC MONOTONICALLY UP (RANDOM TREATMENT EFFECTS) | 165.8 | 64.392 | 126.063 | 43.246 | 39.8 |

## Time

| MODEL | DIC | SD | DEVIANCE | RESIDUAL DEVIANCE | pD |
| --- | --- | --- | --- | --- | --- |
| EMAX (COMMON TREATMENT EFFECTS) | 8871.7 | – | 8857.636 | 8774.819 | 13.9 |
| EMAX (RANDOM TREATMENT EFFECTS) | 165.5 | 62.880 | 126.672 | 43.856 | 39.4 |
| LINEAR (COMMON TREATMENT EFFECTS) | 8927.3 | – | 8913.033 | 8830.216 | 14.1 |
| LINEAR (RANDOM TREATMENT EFFECTS) | 163.2 | 5.595 | 122.822 | 40.005 | 40.1 |
| EXPONENTIAL (COMMON TREATMENT EFFECTS) | 8869.9 | – | 8855.973 | 8773.156 | 13.9 |
| EXPONENTIAL (RANDOM TREATMENT EFFECTS) | 166.5 | 63.675 | 126.462 | 43.646 | 40.1 |
| RESTRICTED CUBIC SPLINE (COMMON TREATMENT EFFECTS; 3 KNOTS) | 8916.7 | – | 8901.789 | 8818.973 | 15.1 |
| RESTRICTED CUBIC SPLINE (RANDOM TREATMENT EFFECTS; 3 KNOTS) | 162.5 | 5.739 | 122.809 | 39.993 | 39.8 |
| NON-PARAMETRIC MONOTONICALLY UP (COMMON TREATMENT EFFECTS) | 8874.4 | – | 8860.158 | 8777.341 | 14.5 |
| NON-PARAMETRIC MONOTONICALLY UP (RANDOM TREATMENT EFFECTS) | 165.5 | 67.240 | 125.760 | 42.944 | 39.9 |

## Weekly time

| MODEL | DIC | SD | DEVIANCE | RESIDUAL DEVIANCE | pD |
| --- | --- | --- | --- | --- | --- |
| EMAX (COMMON TREATMENT EFFECTS) | 8870.4 | – | 8856.409 | 8773.592 | 14.1 |
| EMAX (RANDOM TREATMENT EFFECTS) | 167.0 | 63.313 | 126.674 | 43.857 | 40.6 |
| LINEAR (COMMON TREATMENT EFFECTS) | 8925.1 | – | 8911.477 | 8828.660 | 13.9 |
| LINEAR (RANDOM TREATMENT EFFECTS) | 161.8 | 5.612 | 122.767 | 39.951 | 39.3 |
| EXPONENTIAL (COMMON TREATMENT EFFECTS) | 8870.1 | – | 8856.210 | 8773.394 | 14.0 |
| EXPONENTIAL (RANDOM TREATMENT EFFECTS) | 166.4 | 63.453 | 126.491 | 43.674 | 39.9 |
| RESTRICTED CUBIC SPLINE (COMMON TREATMENT EFFECTS; 3 KNOTS) | 8925.8 | – | 8911.112 | 8828.296 | 14.9 |
| RESTRICTED CUBIC SPLINE (RANDOM TREATMENT EFFECTS; 3 KNOTS) | 162.8 | 5.692 | 122.748 | 39.931 | 40.1 |
| NON-PARAMETRIC MONOTONICALLY UP (COMMON TREATMENT EFFECTS) | 8875.1 | – | 8859.488 | 8776.671 | 15.3 |
| NON-PARAMETRIC MONOTONICALLY UP (RANDOM TREATMENT EFFECTS) | 165.1 | 71.056 | 124.814 | 41.998 | 39.8 |

## Intensity

| MODEL | DIC | SD | DEVIANCE | RESIDUAL DEVIANCE | pD |
| --- | --- | --- | --- | --- | --- |
| EMAX (COMMON TREATMENT EFFECTS) | 8870.6 | – | 8856.437 | 8773.620 | 14.2 |
| EMAX (RANDOM TREATMENT EFFECTS) | 166.6 | 63.130 | 126.088 | 43.272 | 40.0 |
| LINEAR (COMMON TREATMENT EFFECTS) | 8925.5 | – | 8911.314 | 8828.498 | 14.1 |
| LINEAR (RANDOM TREATMENT EFFECTS) | 162.0 | 5.585 | 122.523 | 39.706 | 39.4 |
| EXPONENTIAL (COMMON TREATMENT EFFECTS) | 8870.2 | – | 8856.005 | 8773.188 | 14.1 |
| EXPONENTIAL (RANDOM TREATMENT EFFECTS) | 166.0 | 63.214 | 126.214 | 43.398 | 39.5 |
| RESTRICTED CUBIC SPLINE (COMMON TREATMENT EFFECTS; 3 KNOTS) | 8926.1 | – | 8911.182 | 8828.366 | 15.0 |
| RESTRICTED CUBIC SPLINE (RANDOM TREATMENT EFFECTS; 3 KNOTS) | 161.9 | 5.709 | 122.858 | 40.042 | 39.5 |
| NON-PARAMETRIC MONOTONICALLY UP (COMMON TREATMENT EFFECTS) | 8874.6 | – | 8859.544 | 8776.727 | 14.9 |
| NON-PARAMETRIC MONOTONICALLY UP (RANDOM TREATMENT EFFECTS) | 163.7 | 70.425 | 124.561 | 41.744 | 39.3 |

# deviance plots

## Period


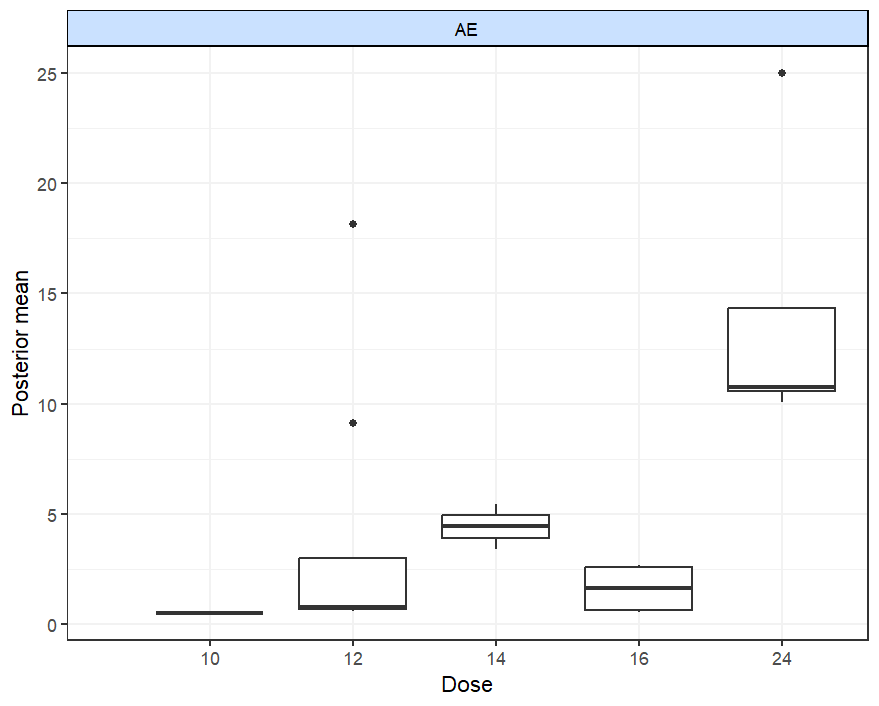


## Frequency


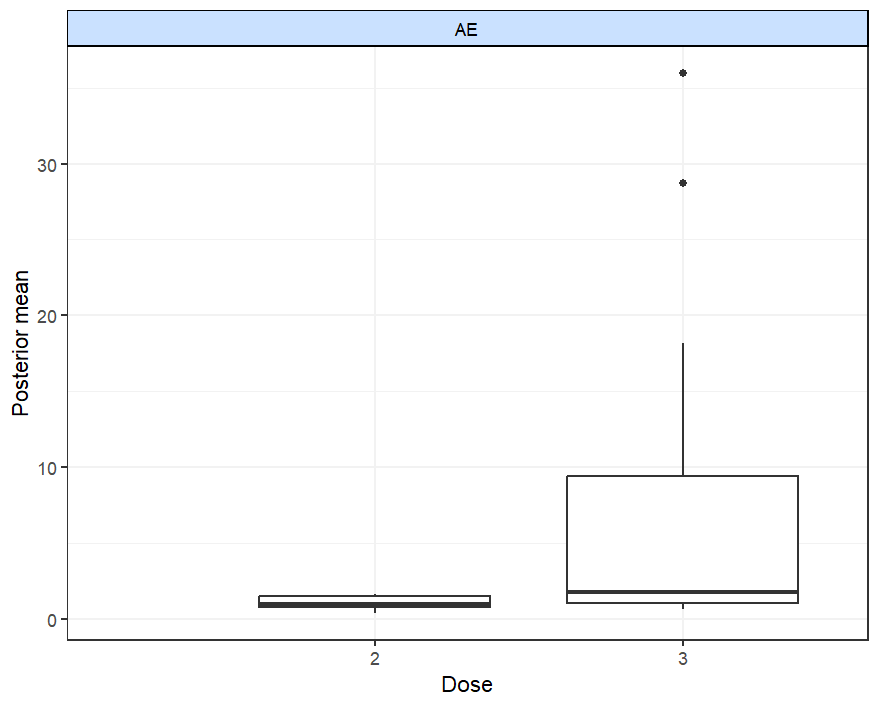


## Time


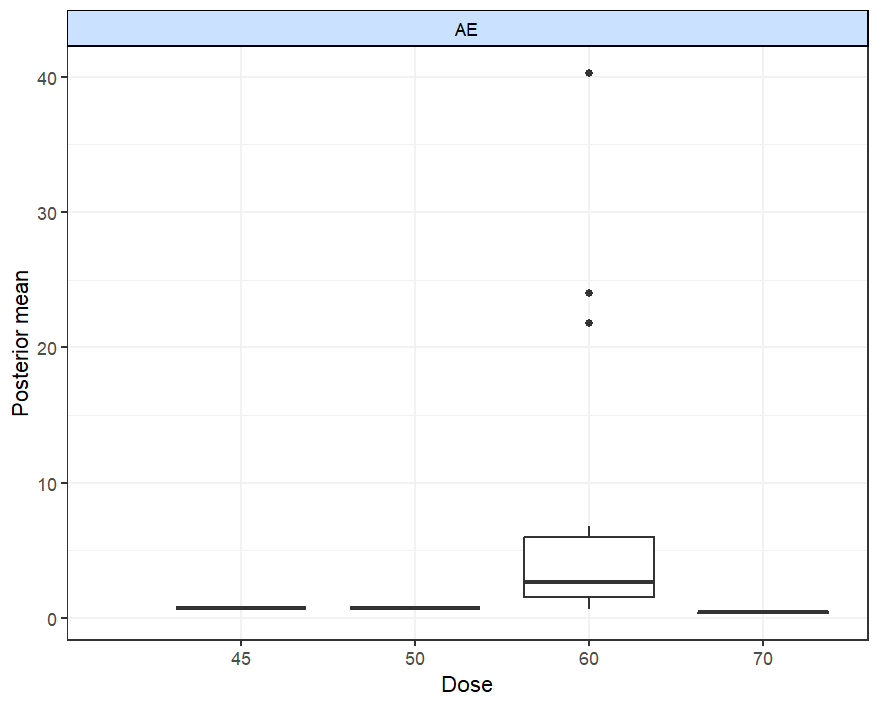


## Weekly time


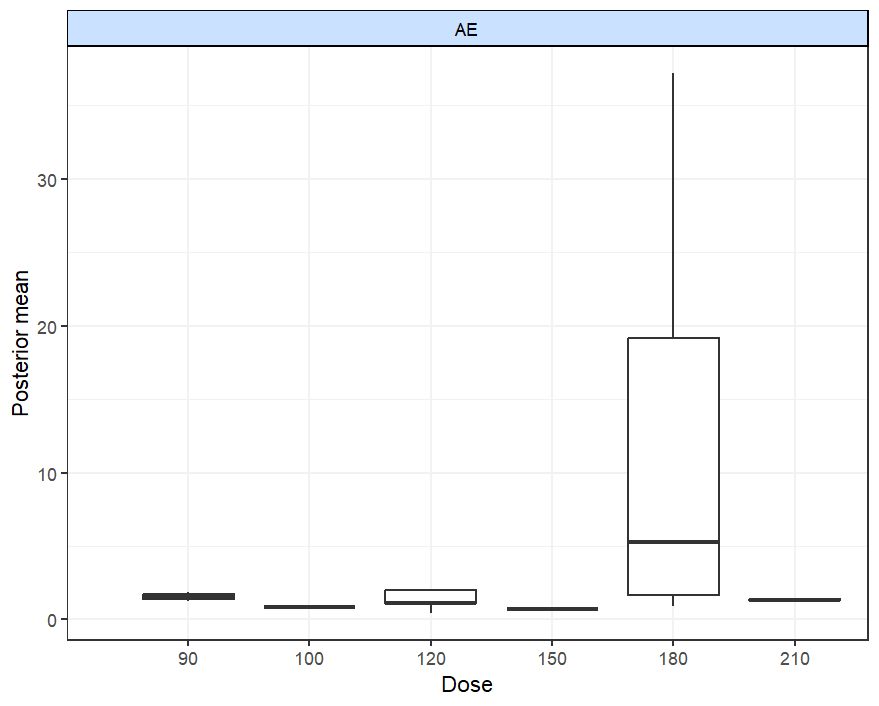


## Intensity


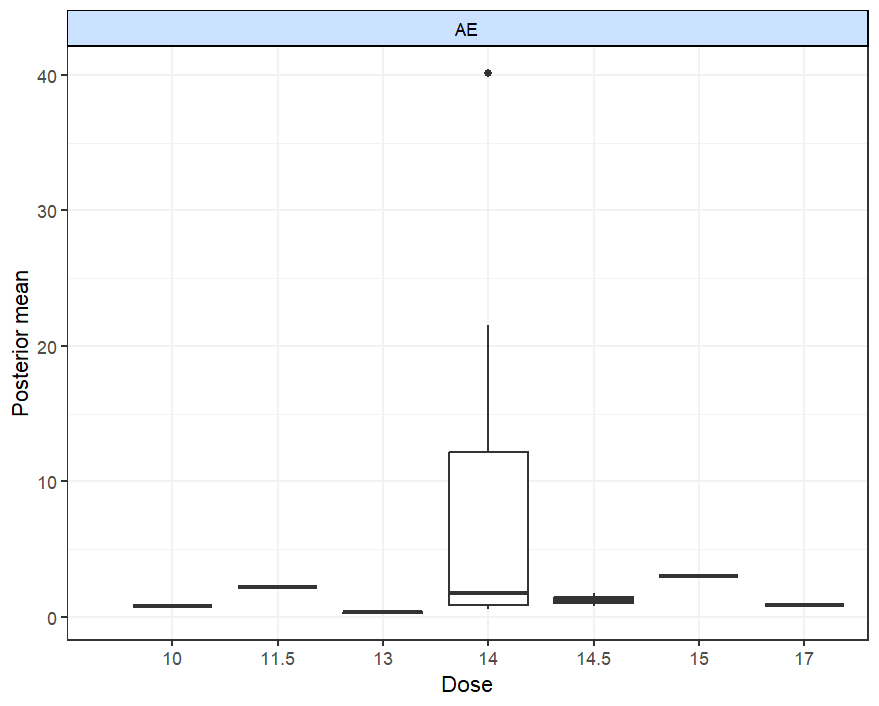


# Model convergence degree

Frequency

| parameter | Rhat (Point est.) | Rhat (Upper CI) |
| --- | --- | --- |
| beta.1[2] | 0.9997 | 1.0001 |
| beta.2[2] | 1.0001 | 1.0012 |
| Deviance | 1.0011 | 1.0034 |
| TotResDev | 1.0011 | 1.0034 |

Period

| parameter | Rhat (Point est.) | Rhat (Upper CI) |
| --- | --- | --- |
| beta.1[2] | 1.0006 | 1.0034 |
| beta.2[2] | 1.0015 | 1.0066 |
| Deviance | 0.9998 | 1.0003 |
| TotResDev | 0.9998 | 1.0003 |

Time

| parameter | Rhat (Point est.) | Rhat (Upper CI) |
| --- | --- | --- |
| beta.1[2] | 1.0006 | 1.0034 |
| beta.2[2] | 1.0015 | 1.0066 |
| Deviance | 0.9998 | 1.0003 |
| TotResDev | 0.9998 | 1.0003 |

Weekly time

| pararmeter | Point est. | Upper C.I. |
| --- | --- | --- |
| beta.1[2] | 0.9999263 | 1.001065 |
| beta.2[2] | 1.0001850 | 1.001367 |
| deviance | 1.0012636 | 1.002592 |
| totresdev | 1.0012636 | 1.002592 |

Intensity

| parameter | Rhat (Point est.) | Rhat (Upper CI) |
| --- | --- | --- |
| beta.1[2] | 1.00195 | 1.00550 |
| beta.2[2] | 1.00089 | 1.00183 |
| Deviance | 1.00352 | 1.01121 |
| TotResDev | 1.00352 | 1.01121 |

# Publication Bias

Table 8 1. Comparison of Unadjusted and Publication Bias-Adjusted Summary Effect Sizes using the Vevea & Hedges Selection Model

| Model Description | Summary Effect Size (SMD) | 95% Confidence Interval (CI) | p-value | τ² (Heterogeneity) |
| --- | --- | --- | --- | --- |
| Standard Random-Effects Meta-Analysis | 0.74 | [0.27, 1.20] | 0.002 | 0.56 |
| Vevea & Hedges Selection Model | 1.65 | [1.08, 2.22] | < 0.001 |  |

## Note: SMD: Standardized Mean Difference; CI: Confidence Interval. The Adjusted Model uses a step-function with p-value cut-points at 0.025 and 0.05 to account for publication bias. The significant Likelihood Ratio Test (p < 0.05) indicates the presence of publication bias, suggesting the Adjusted Model provides a better fit to the data. The estimated τ² reflects the amount of residual heterogeneity.

# PRISMA Checklist

| **Section and Topic** | **Item #** | **Checklist item** | **Location where item is reported** |
| --- | --- | --- | --- |
| **TITLE** | | |  |
| Title | 1 | Identify the report as a systematic review. | Title Page1 |
| **ABSTRACT** | | |  |
| Abstract | 2 | See the PRISMA 2020 for Abstracts checklist. | Page1 |
| **INTRODUCTION** | | |  |
| Rationale | 3 | Describe the rationale for the review in the context of existing knowledge. | INTRODUCTION Page 3 |
| Objectives | 4 | Provide an explicit statement of the objective(s) or question(s) the review addresses. | INTRODUCTION  Page 3 |
| **METHODS** | | |  |
| Eligibility criteria | 5 | Specify the inclusion and exclusion criteria for the review and how studies were grouped for the syntheses. | METHODS, "Eligibility criteria" subsection Page4 |
| Information sources | 6 | Specify all databases, registers, websites, organisations, reference lists and other sources searched or consulted to identify studies. Specify the date when each source was last searched or consulted. | METHODS, "Study selection and search strategies" subsection Page5 |
| Search strategy | 7 | Present the full search strategies for all databases, registers and websites, including any filters and limits used. | METHODS, "Study selection and search strategies" subsection Page5 |
| Selection process | 8 | Specify the methods used to decide whether a study met the inclusion criteria of the review, including how many reviewers screened each record and each report retrieved, whether they worked independently, and if applicable, details of automation tools used in the process. | METHODS, "Study selection and data extraction" subsection Page5 |
| Data collection process | 9 | Specify the methods used to collect data from reports, including how many reviewers collected data from each report, whether they worked independently, any processes for obtaining or confirming data from study investigators, and if applicable, details of automation tools used in the process. | METHODS, "Study selection and data extraction" subsection Page5 |
| Data items | 10a | List and define all outcomes for which data were sought. Specify whether all results that were compatible with each outcome domain in each study were sought (e.g. for all measures, time points, analyses), and if not, the methods used to decide which results to collect. | METHODS, "Eligibility criteria" Page4 |
|  | 10b | List and define all other variables for which data were sought (e.g. participant and intervention characteristics, funding sources). Describe any assumptions made about any missing or unclear information. | METHODS, "Study selection and data extraction Page5 |
| Study risk of bias assessment | 11 | Specify the methods used to assess risk of bias in the included studies, including details of the tool(s) used, how many reviewers assessed each study and whether they worked independently, and if applicable, details of automation tools used in the process. | METHODS, "Risk of bias" subsection Page5 |
| Effect measures | 12 | Specify for each outcome the effect measure(s) (e.g. risk ratio, mean difference) used in the synthesis or presentation of results. | METHODS, "Statistical analysis" Page7 |
| Synthesis methods | 13a | Describe the processes used to decide which studies were eligible for each synthesis (e.g. tabulating the study intervention characteristics and comparing against the planned groups for each synthesis (item #5)). | METHODS, "Study selection and data extraction" Page 4 |
|  | 13b | Describe any methods required to prepare the data for presentation or synthesis, such as handling of missing summary statistics, or data conversions. | METHODS, "Study selection and data extraction" Page4 |
|  | 13c | Describe any methods used to tabulate or visually display results of individual studies and syntheses. | METHODS, "Statistical analysis" Page7 |
|  | 13d | Describe any methods used to synthesize results and provide a rationale for the choice(s). If meta-analysis was performed, describe the model(s), method(s) to identify the presence and extent of statistical heterogeneity, and software package(s) used. | METHODS, "Statistical analysis" Page7 |
|  | 13e | Describe any methods used to explore possible causes of heterogeneity among study results (e.g. subgroup analysis, meta-regression). | METHODS, "Statistical analysis" Page7 |
|  | 13f | Describe any sensitivity analyses conducted to assess robustness of the synthesized results. | RESULTS, "Sensitivity Analysis" Page14 |
| Reporting bias assessment | 14 | Describe any methods used to assess risk of bias due to missing results in a synthesis (arising from reporting biases). | METHODS, "Statistical analysis" Page7 |
| Certainty assessment | 15 | Describe any methods used to assess certainty (or confidence) in the body of evidence for an outcome. | Limitation Page19 |
| **RESULTS** | | |  |
| Study selection | 16a | Describe the results of the search and selection process, from the number of records identified in the search to the number of studies included in the review, ideally using a flow diagram. | RESULTS, "Description of the studies" Page8 |
|  | 16b | Cite studies that might appear to meet the inclusion criteria, but which were excluded, and explain why they were excluded. | RESULTS, ""Description of the studies " Page8 |
| Study characteristics | 17 | Cite each included study and present its characteristics. | RESULTS, "Description of the studies" Page8 |
| Risk of bias in studies | 18 | Present assessments of risk of bias for each included study. | METHODS, "Risk of bias" Page6 |
| Results of individual studies | 19 | For all outcomes, present, for each study: (a) summary statistics for each group (where appropriate) and (b) an effect estimate and its precision (e.g. confidence/credible interval), ideally using structured tables or plots. | RESULTS, "Meta-analysis" Page8 |
| Results of syntheses | 20a | For each synthesis, briefly summarise the characteristics and risk of bias among contributing studies. | RESULTS, "Meta-analysis" Page8-12 |
|  | 20b | Present results of all statistical syntheses conducted. If meta-analysis was done, present for each the summary estimate and its precision (e.g. confidence/credible interval) and measures of statistical heterogeneity. If comparing groups, describe the direction of the effect. | RESULTS, "Meta-analysis" Page8-16 |
|  | 20c | Present results of all investigations of possible causes of heterogeneity among study results. | RESULTS, "Meta-analysis" Page8-16 |
|  | 20d | Present results of all sensitivity analyses conducted to assess the robustness of the synthesized results. | RESULTS, "Sensitivity Analysis" Page14 |
| Reporting biases | 21 | Present assessments of risk of bias due to missing results (arising from reporting biases) for each synthesis assessed. | RESULTS, "Publication Bias Analysis" Page14 |
| Certainty of evidence | 22 | Present assessments of certainty (or confidence) in the body of evidence for each outcome assessed. | Limitation Page19 |
| **DISCUSSION** | | |  |
| Discussion | 23a | Provide a general interpretation of the results in the context of other evidence. | DISCUSSION (First paragraph) Page15 |
|  | 23b | Discuss any limitations of the evidence included in the review. | DISCUSSION, "Limitations" Page19 |
|  | 23c | Discuss any limitations of the review processes used. | DISCUSSION, "Limitations" Page19 |
|  | 23d | Discuss implications of the results for practice, policy, and future research. | DISCUSSION, "Limitations" Page19 |
| **OTHER INFORMATION** | | |  |
| Registration and protocol | 24a | Provide registration information for the review, including register name and registration number, or state that the review was not registered. | METHODS, "Experimental Approach to the Problem" Page4 |
|  | 24b | Indicate where the review protocol can be accessed, or state that a protocol was not prepared. | METHODS, "Experimental Approach to the Problem" Page4 |
|  | 24c | Describe and explain any amendments to information provided at registration or in the protocol. | None |
| Support | 25 | Describe sources of financial or non-financial support for the review, and the role of the funders or sponsors in the review. | None |
| Competing interests | 26 | Declare any competing interests of review authors. | None |
| Availability of data, code and other materials | 27 | Report which of the following are publicly available and where they can be found: template data collection forms; data extracted from included studies; data used for all analyses; analytic code; any other materials used in the review. | RESULTS, "Description of the studies" Page 8 |

## **Prisma abstract checklist**

| **Section and Topic** | **Item #** | **Checklist item** | **Reported (Yes/No)** |
| --- | --- | --- | --- |
| **TITLE** | | |  |
| Title | 1 | Identify the report as a systematic review. | Yes |
| **BACKGROUND** | | |  |
| Objectives | 2 | Provide an explicit statement of the main objective(s) or question(s) the review addresses. | Yes |
| **METHODS** | | |  |
| Eligibility criteria | 3 | Specify the inclusion and exclusion criteria for the review. | Yes |
| Information sources | 4 | Specify the information sources (e.g. databases, registers) used to identify studies and the date when each was last searched. | Yes |
| Risk of bias | 5 | Specify the methods used to assess risk of bias in the included studies. | Yes |
| Synthesis of results | 6 | Specify the methods used to present and synthesise results. | Yes |
| **RESULTS** | | |  |
| Included studies | 7 | Give the total number of included studies and participants and summarise relevant characteristics of studies. | Yes |
| Synthesis of results | 8 | Present results for main outcomes, preferably indicating the number of included studies and participants for each. If meta-analysis was done, report the summary estimate and confidence/credible interval. If comparing groups, indicate the direction of the effect (i.e. which group is favoured). | Yes |
| **DISCUSSION** | | |  |
| Limitations of evidence | 9 | Provide a brief summary of the limitations of the evidence included in the review (e.g. study risk of bias, inconsistency and imprecision). | Yes |
| Interpretation | 10 | Provide a general interpretation of the results and important implications. | Yes |
| **OTHER** | | |  |
| Funding | 11 | Specify the primary source of funding for the review. | No |
| Registration | 12 | Provide the register name and registration number. | No |

# TIDieR scale analysis

| Study | 1 | 2 | 3 | 4 | 5 | 6 | 7 | 8 | 9 | 10 | 11 | 12 |
| --- | --- | --- | --- | --- | --- | --- | --- | --- | --- | --- | --- | --- |
| Bento，et al.(2012) | Y | Y | P | P | N | P | P | Y | N | N | N | N |
| Bergamin, et al. (2013) | Y | Y | P | Y | P | Y | Y | Y | P | N | P | Y |
| Bocalini, et al. (2008) | Y | Y | P | P | P | Y | Y | Y | P | N | N | Y |
| Chen Y, et al. (2024) | Y | Y | Y | Y | P | Y | Y | Y | Y | N | P | Y |
| Ferreira, et al. (2022) | Y | Y | P | Y | N | P | P | Y | N | N | N | P |
| Martínez-Rodríguez, et al. (2022) | Y | Y | P | Y | N | P | P | Y | N | N | N | P |
| Moreira, et al. (2020) | Y | Y | P | Y | N | P | P | Y | P | N | N | P |
| Oh, et al. (2015) | Y | Y | N | P | N | P | N | N | N | N | N | N |
| Takeshima, et al. (2002) | Y | Y | P | Y | N | P | P | Y | N | N | N | N |
| Taunton, et al. (1996) | Y | Y | N | P | N | Y | N | Y | N | Y | N | N |
| Tsourlou, et al. (2006) | Y | Y | Y | Y | N | Y | N | Y | N | Y | N | N |
| Graef, et al. (2010) | Y | Y | Y | Y | N | Y | Y | Y | N | Y | N | N |
| Kim, et al. (2013) | Y | Y | N | Y | N | Y | P | N | Y | Y | N | N |

1: Provide the name or a phrase that describes the intervention.; 2: Describe any rationale, theory, or goal of the elements essential to the intervention.; 3: Materials: Describe any physical or informational materials used in the intervention, including those provided to participants or used in intervention delivery or in training of intervention providers. Provide information on where the materials can be accessed (e.g. online appendix, URL).; 4: Procedures: Describe each of the procedures, activities, and/or processes used in the intervention, including any enabling or support activities.; 5: For each category of intervention provider (e.g. psychologist, nursing assistant), describe their expertise, background and any specific training given.; 6: Describe the modes of delivery (e.g. face-to-face or by some other mechanism, such as internet or telephone) of the intervention and whether it was provided individually or in a group.; 7: Describe the type(s) of location(s) where the intervention occurred, including any necessary infrastructure or relevant features.; 8: Describe the number of times the intervention was delivered and over what period of time including the number of sessions, their schedule, and their duration, intensity or dose.; 9: If the intervention was planned to be personalized, titrated or adapted, then describe what, why, when, and how.; 10: If the intervention was modified during the course of the study, describe the changes (what, why, when, and how).; 11: Planned: If intervention adherence or fidelity was assessed, describe how and by whom, and if any strategies were used to maintain or improve fidelity, describe them.12.Actual: If intervention adherence or fidelity was assessed, describe the extent to which the intervention was delivered as planned. Y for Fully report, P for Partially reported, N for Not reported.

# PEDro Scale Evaluation

| Study | 1 | 2 | 3 | 4 | 5 | 6 | 7 | 8 | 9 | 10 | 11 | Total |
| --- | --- | --- | --- | --- | --- | --- | --- | --- | --- | --- | --- | --- |
| Bento et al. (2012) | Y | Y | N | Y | N | N | N | Y | N | Y | Y | 6 |
| Bergamin et al. (2013) | Y | Y | N | Y | N | N | Y | Y | Y | Y | Y | 7 |
| Bocalini et al. (2008) | Y | Y | N | Y | N | N | N | Y | N | Y | Y | 6 |
| Chen et al. (2024) | Y | Y | N | Y | N | N | Y | Y | Y | Y | Y | 7 |
| Ferreira et al. (2022) | Y | Y | Y | Y | N | N | Y | N | Y | Y | Y | 7 |
| Martínez-Rodríguez et al. (2022) | Y | Y | N | Y | N | N | N | Y | N | Y | Y | 5 |
| Moreira et al. (2020) | Y | Y | N | Y | N | N | Y | N | Y | Y | Y | 6 |
| Oh et al. (2015) | Y | Y | N | Y | N | N | Y | Y | Y | Y | Y | 7 |
| Takeshima et al. (2002) | Y | Y | N | Y | N | N | Y | Y | N | Y | Y | 6 |
| Taunton et al. (1996) | Y | Y | N | Y | N | N | N | N | N | Y | Y | 4 |
| Tsourlou et al. (2006) | Y | N | Y | N | N | Y | N | Y | Y | Y | N | 6 |
| Graef et al. (2010) | Y | Y | N | Y | N | Y | N | Y | Y | Y | N | 6 |
| Kim et al. (2013) | Y | N | Y | N | N | N | N | N | Y | Y | N | 4 |

1. eligibility criteria were specified 2. subjects were randomly allocated to groups (in a crossover study, subjects were randomly allocated an order in which treatments were received) 3. allocation was concealed 4. the groups were similar at baseline regarding the most important prognostic indicators 5. there was blinding of all subjects 6. there was blinding of all therapists who administered the therapy 7. there was blinding of all assessors who measured at least one key outcome 8. measures of at least one key outcome were obtained from more than 85% of the subjects initially allocated to groups 9. all subjects for whom outcome measures were available received the treatment or control condition as allocated or, where this was not the case, data for at least one key outcome was analysed by “intention to treat”. 10. the results of between-group statistical comparisons are reported for at least one key outcome 11. the study provides both point measures and measures of variability for at least one key outcome Y for yes, N for no
